# Effectiveness of case finding strategies for COPD in primary care: a systematic review and meta-analysis

**DOI:** 10.1038/npjpcrm.2015.56

**Published:** 2015-08-27

**Authors:** Shamil MM Haroon, Rachel E Jordan, Joanne O’Beirne-Elliman, Peymane Adab

**Affiliations:** 1 Public Health, Epidemiology & Biostatistics, School of Health and Population Sciences, University of Birmingham, Edgbaston, Birmingham, UK

## Abstract

**Background:**

Chronic obstructive pulmonary disease (COPD) is widely underdiagnosed, but the most effective approach for identifying these patients is unknown.

**Aims:**

The aim of this study was to summarise and compare the effectiveness of different case finding approaches for undiagnosed COPD in primary care.

**Methods:**

A systematic review of primary studies of any design evaluating case finding strategies for COPD in primary care among individuals aged ⩾35 years with no prior diagnosis was conducted. Medline, Embase and other bibliographic databases were searched from 1997 to 2013, and methodological quality was assessed using standard tools. Results were described and meta-analysis of the uptake and yield from different approaches was performed where there was sufficient homogeneity.

**Results:**

Three randomised controlled trials (RCTs), 1 controlled trial and 35 uncontrolled studies were identified that assessed the identification of new cases of COPD through systematic case finding. A range of approaches were used including pre-screening with questionnaires (*n*=13) or handheld flow meters (*n*=5) or direct invitation to diagnostic spirometry (*n*=30). Overall, any approach identified more undiagnosed COPD compared with usual care. Targeting those at higher risk (e.g., smokers) and pre-screening (e.g., using questionnaires) is likely to increase the yield. However, studies were heterogeneous and were limited by a lack of comparison groups, inadequate reporting and diversity in the definition of COPD, which limited our ability to draw firm conclusions.

**Conclusions:**

There is extensive heterogeneity among studies evaluating case finding strategies for COPD, with few RCTs. Well-conducted RCTs comparing case finding approaches are needed to identify the most effective target population, recruitment strategy and screening tests, using a clinical definition of COPD, and addressing the limitations highlighted in this review. There is also a need to evaluate the impact of case finding on clinical care and patient outcomes.

## Introduction

Chronic obstructive pulmonary disease (COPD) is the third leading cause of mortality,^1^ an important cause of disability^2^ and a source of significant healthcare expenditure.^3^ However, up to 70–90% of the disease burden remains undiagnosed.^4^ People with undiagnosed COPD often under-recognise the significance of their symptoms,^5^ and there is poor awareness of the condition among the general public.^6,7^ Clinicians in primary care also frequently miss opportunities to diagnose COPD.^8^ Furthermore, a higher prevalence of undiagnosed COPD has been associated with higher rates of COPD-related hospitalisations,^9^ with diagnosis often being made during an acute hospital admission following an exacerbation.^10^ There is now a policy drive to identify undiagnosed COPD earlier in the course of the disease.^11^ However, the optimal strategy for achieving this remains unknown.^12^

A systematic review published in 2008 evaluated the effectiveness of population-based screening for COPD using spirometry but concluded that this would identify many asymptomatic individuals with mild-to-moderate airflow obstruction, for whom there are limited therapeutic options.^13^ However, since then there have been a large number of studies of potentially more efficient approaches, including the use of screening questionnaires^14^ and handheld flow meters (e.g., Piko-6, Longmont, CO, USA or COPD-6, Buckingham, UK)^15^ and more studies seeking individuals with clinical symptoms.

We report a systematic review to identify and compare the effectiveness (yield) of alternative case finding approaches for COPD in primary care.

## Materials and methods

### Protocol and registration

The protocol for this review was previously published^16^ and registered on the PROSPERO register of systematic reviews (CRD42012002074).^17^

### Eligibility criteria

As RCTs were known to be rare, primary studies of any design were sought that were conducted in primary care (including general practices and community pharmacies), recruited individuals aged ⩾35 years with no prior diagnosis of COPD (or provided sufficient data to separate out subjects with previously known and newly diagnosed COPD) and aimed to detect undiagnosed COPD confirmed by spirometry. Eligible screening tests included questionnaires, clinical examination, handheld flow meters, peak flow meters, decision aids/risk prediction models and chest radiography, either alone or in combination.

Note that there is no clear definition of the difference between case finding and screening. Some authors define screening as identifying asymptomatic patients and case finding as identifying patients with clinical disease, although the definition provided by the UK National Screening Committee includes structured case finding within screening.^18^ In this review, we sought studies that defined COPD using spirometric criteria, and draw particular attention to the studies in which the definition of COPD additionally incorporated clinical symptoms.

### Search strategy

We searched Medline, Embase, CINAHL, Cochrane Central Register of Controlled Trials (CENTRAL), Database of Abstracts of Reviews of Effects, HTA Database and NHS Economic Evaluations Database from 1 March 2012 for the previous 15 years and performed an updated search in Medline and Embase up to December 2013. To identify grey literature, searches were also performed on Google Scholar, Turning Research into Practice, HTAi Vortal and DogPile, limited to the first 100 articles per search. No language restrictions were applied (Supplementary Table S1).

### Study selection and data extraction

Titles and abstracts were screened independently by two reviewers. Full-text papers were obtained for all potentially relevant studies, and the eligibility criteria were applied independently, with disagreements resolved through discussion. Data were extracted on the characteristics of the selected population, approaches to recruitment, method of screening, number of participants newly diagnosed with COPD and numbers who were eligible, participated in screening and underwent diagnostic spirometry. In cases in which these data were not provided, we used information provided in the paper to derive them.

### Methodological quality assessment

Included studies were assessed independently by two reviewers against criteria adapted from the QUADAS-1^19^ and QUADAS-2^20^ checklists, and the Cochrane risk of bias assessment tool for RCTs.^21^ Disagreements were resolved through discussion.

### Statistical analysis

Results from comparative studies were described but not suitable for synthesis. We therefore used data from individual study arms of the trials together with the non-comparative studies to summarise uptake of tests and yields. Where there was sufficient methodological homogeneity, these were combined using random-effects meta-analyses. Forest plots were constructed to explore between-study heterogeneity in the yield, including differences in population characteristics, screening tests, diagnostic criteria and study design. All analyses were performed using Stata version 13.1 (Stata-Corp, College Station, TX, USA) and StatsDirect version 2.7.9 (Altrincham, Cheshire, UK).

## Results

### Study selection

**Figure 1:**
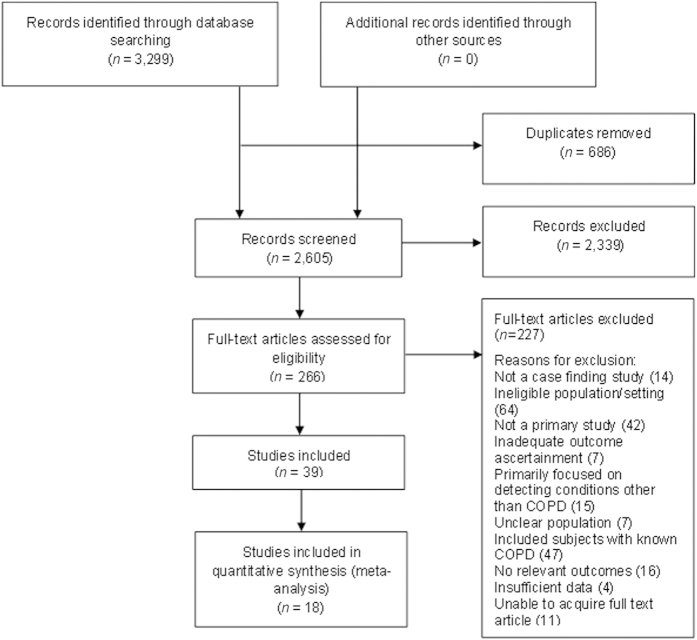
Article selection.

After removing duplicates, 2,605 citations were identified and 266 full-text articles were assessed for eligibility (Figure 1). A total of 39 studies were finally selected, from which 18 were included in meta-analyses. Studies that did not exclude patients with previously known COPD, or those that did not provide sufficient data to separate out new from existing diagnoses of COPD, were excluded.

### Overall study characteristics

There were 2 individual RCTs, 1 cluster RCT, 1 non-randomised controlled trial, 25 single-arm before-after studies and 10 cross-sectional test accuracy studies (Supplementary Table S2). Most of them were conducted in general practices (*n*=34), two in community pharmacies and the remaining studies in either a health screening clinic (*n*=1) or unclear primary care setting (*n*=2). They evaluated the use of screening questionnaires (*n*=13), handheld flow meters (*n*=5) and direct invitation for diagnostic spirometry (*n*=30). No studies evaluating other screening tests met the inclusion criteria.

### Comparative studies

The comparative studies were highly heterogeneous, with major differences in trial design and comparators (Table 1). An RCT of nurse-led case finding among four general practices in Australia showed that using written invitations to attend spirometry led to 2.3% (95% confidence interval (CI) 0.7 to 3.9%) higher yield of new cases than usual care.^22^ A cluster RCT in the Netherlands found that a practice-managed approach was more effective than patient self-scoring of a respiratory screening questionnaire before spirometry assessment (difference in yield 0.9% (0.5 to 1.3%)).^23^ Our own pilot trial comparing postal versus opportunistic screening using a respiratory questionnaire before diagnostic spirometry indicated that the former might lead to higher yield.^24^ However, the study lacked sufficient power to detect significant differences. Finally, a non-randomised trial found that offering spirometry to patients attending primary care suspected by their GP to have COPD resulted in a significantly higher yield than mass invitation to people with chronic respiratory symptoms (difference 18.6% (95% CI 12.6 to 24.6%)).^25^ These comparative studies suffered from problems of randomisation,^22,25^ inadequate blinding of assessors to intervention arms^22–25^ and insufficient clarity about whether the populations in trial arms were comparable^22,23^ (Supplementary Table S3). There was too much heterogeneity in their design and outcomes to combine their results.

### Estimating the effect of different case finding approaches using all data from uncontrolled studies and single arms of trials

#### Methodological quality

**Figure 2:**
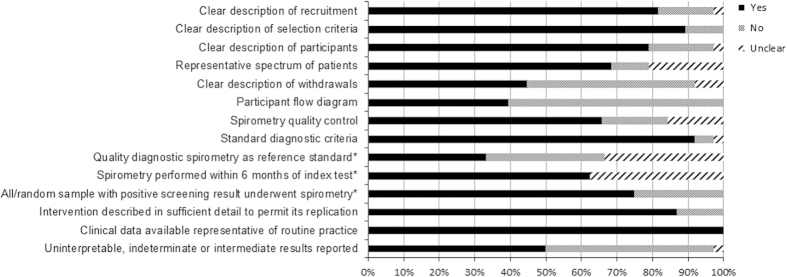
Quality assessment. '*' indicates only studies evaluating screening questionnaires and/or handheld flow meters.

The majority of included studies were uncontrolled single-arm studies and were highly heterogeneous. They generally provided a clear description of recruitment, selection criteria, characteristics of screened and clinically assessed participants and how spirometry was performed. Most of the studies used standard diagnostic criteria for COPD or airflow obstruction as the outcome. However, they often failed to report the complete eligible population, withdrawals, uninterpretable and indeterminate test results, participant flow or spirometry quality control procedures (Figure 2, Supplementary Tables S4a,b).

#### Recruitment and population selection

Participants were actively recruited through postal invitations, telephone calls and advertisements (*n*=17 studies), were opportunistically invited at primary care consultations (*n*=17) or were recruited through a combination of both approaches (*n*=2; Table 2, Supplementary Table S2). Age (usually ⩾40 years) and a positive smoking history were the main eligibility criteria, although more than half of the studies included never smokers. A small number of studies specified additional eligibility criteria including the presence of respiratory symptoms^26–29^ or recent acute respiratory infections.^30^

#### Screening tests and diagnosis of COPD

Strategies for targeting those at high risk included using basic patient characteristics only (e.g., age and smoking status (*n*=30)), use of respiratory screening questionnaires (*n*=13) and administration of handheld flow meters (*n*=5) before diagnostic spirometry. Only nine studies included a clinical component to the case definition of COPD. All other studies used a purely physiological definition of COPD based on airflow limitation, most commonly a forced expiratory volume in 1 s to forced vital capacity ratio of <70% (FEV_1_/FVC<0.7; *n*=30). In addition, 13 used pre-bronchodilator spirometry for diagnosis, although the yields did not seem to significantly differ according to the use of pre- or post-bronchodilator testing.

#### Yield of new COPD cases with different approaches

Table 2 summarises the characteristics and yield (proportion of new cases) of all of the included studies for each case finding approach. With direct invitation for diagnostic spirometry, the overall proportion of all eligible subjects newly diagnosed with COPD (as defined by each study) ranged from 1.7 to 30.5% (18 studies; Table 2, Supplementary Tables S5 and S6). Most of them had mild-to-moderate disease, although in some studies a proportion (up to 37.2%) of subjects had severe disease, which may have been related to the inclusion of a higher proportion of current smokers and patients with respiratory symptoms. Studies with highest yields were mainly test accuracy studies (which generally had higher methodological quality and possibly had more robust methods for inviting, assessing and following up participants) and those including only symptomatic patients.

Among 13 studies that tested screening questionnaires before diagnostic spirometry (Table 2, Supplementary Table S7), the COPD Diagnostic Questionnaire^14^ (also referred to as the International Primary Airways Group Questionnaire and the Respiratory Health Screening Questionnaire) was most widely evaluated (*n*=5 studies). Overall, new cases of COPD ranged from 0.4 to 22.3% of those eligible, again with the highest yields found in test accuracy studies.

Five (mostly test accuracy) studies evaluated screening with handheld flow meters before diagnostic spirometry (Table 2, Supplementary Tables S8 and S9). Overall, the yield ranged from 6 to 20% of those eligible, and all but one recruited patients opportunistically.

Because of the methodological heterogeneity, it was not possible to combine studies or draw firm conclusions, although incorporating an initial screening test before diagnostic assessment resulted in generally higher yield (percentage diagnosed with COPD out of all those referred for spirometry was 19–94% for handheld flow meters, 14.3–42.1% with screening questionnaires and 4.1–40.2% with no pre-screening).

### Exploring the effect of different target populations and recruitment strategies

We combined the results of studies including ever smokers, and a further subset among those already reporting respiratory symptoms because they appeared to be relatively homogeneous.

### Uptake of screening/diagnostic tests

Opportunistic invitation of ever smokers at routine primary care attendances was associated with a significantly higher uptake of spirometry (97% (95% CI 90 to 100%); *n*=5 studies^28,31–34^) than actively inviting ever-smoking patients by post (47% (16 to 80%); *n*=6 studies^22,30,35–38^), although CIs were wide (which may reflect heterogeneity in the populations being studied). Meta-analysis (*n*=4 studies^23,24,35,39^) showed an average response to postal questionnaires of 30% (95% CI 20 to 41%). There were insufficient data to estimate the response when questionnaires were distributed opportunistically at primary care attendances and to estimate the overall yield from either approach.

### Target populations

**Figure 3:**
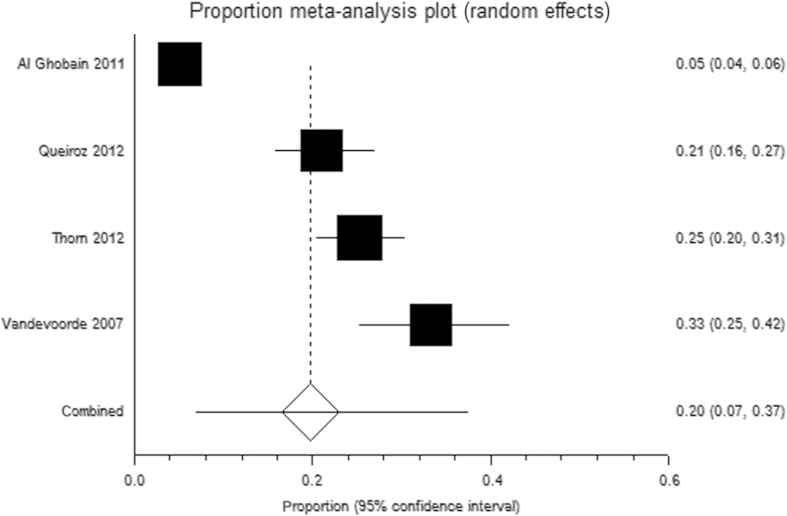
Random-effects meta-analysis of the proportion of eligible ever smokers diagnosed with COPD when opportunistically invited for spirometry (restricted to studies that defined airflow obstruction as FEV_1_/FVC<0.7).

From four similar studies, in which ever smokers aged ⩾40 years were opportunistically invited directly for diagnostic spirometry, 20% (95% CI 7 to 37%;^31,33,40,41^) were diagnosed with airflow obstruction (Figure 3), although CIs were wide and not all would necessarily have clinical disease. Restricting recruitment to ever smokers attending primary care and reporting a history of respiratory symptoms/infections provided a higher yield of 32% (95% CI 21 to 44%; *n*=3 studies^27,28,42^), although again these CIs were wide.

## Discussion

### Main findings

This review incorporated evidence from 39 primary studies. The few comparative studies had a number of methodological limitations and were highly heterogeneous. They suggested that active case finding was likely to identify a greater number of patients with undiagnosed COPD compared with usual care,^22^ that scoring of screening questionnaires before diagnostic assessment should be practice-led rather than patient-led^23^ and that assessment of patients clinically suspected to have COPD is likely to result in higher yield than widespread invitation of the general public for screening.^25^ The remaining studies were non-comparative, with significant limitations in study design and the reporting of eligible populations, withdrawals, indeterminate results and spirometry quality control procedures. The lack of direct comparisons and the heterogeneity in populations, study design, definition of COPD and methods for estimating the yield limited our ability to draw firm conclusions about the most effective case finding approach.

However, indirect comparisons suggested that uptake for spirometry was higher when patients were invited opportunistically at routine primary care visits than with active postal invitation, and response to mailed screening questionnaires is likely to be ~30%. Studies that opportunistically screened ever smokers with a history of respiratory symptoms seemed to have a higher yield (although not statistically significant) than opportunistic studies among ever smokers in which symptoms were not considered. Yields from different approaches were highly variable, although pre-screening using either a questionnaire or handheld flow meter seemed to achieve a higher yield than direct invitation for diagnostic assessment. However, no studies had directly compared questionnaires with the use of handheld devices, nor considered respective costs. Finally, case finding is likely to uncover a significant burden of disease, and although the majority of this will be of mild-to-moderate severity a notable proportion (>10% in several studies) will have severe airflow obstruction.

### Interpretation of findings in relation to previously published work

The UK National Screening Committee recently recommended against screening for COPD because of a lack of relevant RCTs, and insufficient evidence on the optimal approach and the benefits of early treatment.^12^ They also concluded that opportunistic case finding among symptomatic individuals with more developed COPD was likely to be cost-effective and that it should continue. An older systematic review concluded that mass invitation for diagnostic spirometry (without prior pre-screening) could not be recommended, as large numbers of assessments would be needed to prevent a single COPD exacerbation, and would largely unveil patients with asymptomatic airflow obstruction for whom there are limited therapeutic options.^13^ Our findings concur with these points but also highlight several important methodological limitations that should be addressed in future trials evaluating case finding strategies. Furthermore, we found that although the majority of new patients had mild disease (which is arguably when secondary prevention may be most effective) a substantial proportion of those diagnosed are likely to have moderate-to-severe disease, with potential to benefit from evidence-based therapies.^43^

Jithoo *et al*.^44^ recently compared alternative case finding strategies, concluding that peak flow meters were a cost-effective screening test for COPD. However, the peak expiratory flow rate was derived from quality-controlled spirometry measurements and may not necessarily reflect the accuracy of peak flow meters used in everyday clinical practice. Our search strategy did not identify studies directly evaluating peak flow meters for case finding. RCTs comparing the effectiveness of screening with peak flow meters against other screening tests such as questionnaires and handheld flow meters should therefore be considered.

### Strengths and limitations of this study

We performed an extensive review of the literature and incorporated detailed evidence on 39 primary studies. However, there were very few RCTs, and heterogeneity among those identified precluded combining them. We therefore mainly relied on indirect comparisons, where potential bias arising from differences in study design and population characteristics made their combination inappropriate. Missing information on the size of the eligible population limited estimates of the uptake of screening and could potentially bias estimates obtained from meta-analyses. Furthermore, many of the combined results had wide confidence intervals and should be interpreted with caution. Unfortunately, with so few RCTs and so many single-arm studies, it was not possible to undertake the usual statistical analyses to test for publication bias. However, the search strategy was very comprehensive, and thus relevant RCTs are unlikely to have been missed.

Another important limitation was that most studies used a physiological definition of COPD based on spirometry, without clinical confirmation (ascertaining the presence of a compatible clinical history, including the presence of relevant symptoms). This is likely to overestimate the effectiveness (yield) of case finding, as a previous analysis undertaken by our group^45^ found that only about half of the individuals with undiagnosed airflow obstruction reported symptoms compatible with a clinical diagnosis of COPD. Identification of individuals with asymptomatic airflow obstruction is problematic in the absence of evidence-based recommendations to guide their management.^13^ In addition, the use of the fixed ratio for FEV_1_/FVC is controversial and many people believe this may also lead to overdiagnosis.^46^ Conversely, patients with undiagnosed COPD may underreport symptoms, as they often adapt to them over time until the quality-of-life is markedly affected, which may contribute to diagnostic delay.^47^

### Implications for future research, policy and practice

Case finding through any approach is likely to uncover a substantial number of patients with undiagnosed COPD. With the current evidence, it is unclear whether opportunistic or active case finding results in a higher overall yield, although targeting patients with a history of respiratory symptoms or infections might improve the case finding yield. Pre-screening using either a questionnaire such as the COPD Diagnostic Questionnaire or handheld flow meter may reduce the number of diagnostic assessments needed compared with directly inviting all high-risk patients for diagnostic spirometry. However, it is unclear whether pre-screening with a handheld device would reduce the overall number of clinical contacts or improve the use of healthcare resources. Although the majority of patients identified through case finding will have mild-to-moderate disease, around one in five is likely to have severe disease with potential to benefit from being identified.

At the moment, opportunistic case finding is recommended as a cost-effective strategy by the National Institute for Health and Care Excellence (NICE).^48^ Our review concludes that focusing case finding opportunistically on those at high risk (based on age and previous smoking history), and reporting relevant clinical symptoms, is likely to improve the efficiency of case finding. However, there is insufficient evidence from well-conducted comparative studies to suggest which wider screening approach would result in higher yield, and there is insufficient evidence to support a more active approach to case finding.

No further information is needed from single-arm non-controlled studies. Future studies should directly compare approaches, particularly addressing how yield could be further increased by targeting those at highest risk (e.g., by using risk prediction models), considering alternative recruitment strategies (opportunistic versus a more active approach for inviting those at high risk) and alternative screening tests (e.g., questionnaires, handheld flow meters, peak flow meters and risk prediction models, alone and in combination).

A definition of COPD should be used that is standardised and aligns with current recommendations, requiring clinical confirmation in addition to airflow limitation.^11^ They should also report withdrawals and indeterminate results and present this in a participant flow chart, including a description of the size and characteristics of the eligible population and those who received a screening and diagnostic assessment. They should also use strict quality control procedures for spirometry to avoid misdiagnosis. The findings of these studies will be important to enable economic evaluations of alternative case finding strategies to aid policymakers to make appropriate decisions on the most cost-effective approach.

### Conclusions

There is extensive heterogeneity and few RCTs among studies evaluating case finding strategies for COPD in primary care, and it remains unclear as to which approach has the higher overall yield. Observational studies suggest that targeting specific sub-groups such as ever smokers with a history of respiratory symptoms may be more efficient, and using screening tests such as questionnaires may reduce the number of diagnostic assessments needed to identify a patient with COPD. High-quality RCTs are needed to make direct comparisons of alternative case finding strategies, including the target population, recruitment strategy and screening tests, taking into consideration the methodological limitations of previous studies highlighted in this review. There is currently inadequate evidence of effectiveness or cost-effectiveness for recommending any particular approach to case finding in primary care. There is also a need to evaluate the impact of case finding on clinical care and patient outcomes before firm recommendations can be made.

## Figures and Tables

**Table 1:** Comparative studies

*Study*	*Study design and setting*	*Eligibility criteria*	*Intervention*	*Comparator*	*COPD definition*	*COPD*^a^*/eligible*^b^	*Limitations*
Bunker 2009^22^	RCT: nurse-led case finding using spirometry versus usual care in four practices (recruitment dates not reported).	Inclusion criteria: ever smokers aged 40–80 years. Exclusion criteria: known diagnosis of COPD, cognitive impairment, non-English speaking and <2 visits to practice in the preceding year.	Case finding arm: spirometry (unclear whether pre- or post-BD) performed by practice nurses.	Routine care.	FEV_1_/FVC<70% (unclear whether pre- or post-BD).	Case finding: 10/400 (2.5%) Routine care: 1/408 (0.2%). Difference in yield=2.3% (95% CI 0.7 to 3.9%).	Method of randomisation was not described. Inadequate outcome ascertainment. Outcome assessors were not blinded. Unclear whether intervention groups were comparable.
Dirven 2013^23^	Cluster RCT: patient versus practice-managed scoring of a screening questionnaire in 16 general practices from May to September 2012.	Inclusion criteria: age 40–70 years. Exclusion criteria: previous diagnosis of asthma, COPD or significant lung disease. Patients using oxygen supplementation and those with impaired mobility were also excluded.	Patient-managed arm Stage 1: patients were mailed the Respiratory Health Screening Questionnaire, self-calculated risk of COPD and advised to consult GP if score was >19.5. Stage 2: post-BD spirometry for subjects scoring >19.5.	Practice-managed arm Stage 1: patients were mailed the questionnaire, but scoring was performed by healthcare staff. Stage 2: post-BD spirometry on subjects scoring >19.5.	Post-BD FEV_1_/FVC<0.7 and physician’s clinical evaluation.	Patient managed: 25/6393 (0.4%). Practice managed: 48/3715 (1.3%). Difference in yield=0.9% (95% CI 0.5 to 1.3%).	Unclear whether intervention groups were comparable (although practices were stratified by socioeconomic status). Unclear whether outcome assessors were blinded.
Haroon 2013^16^	RCT: active versus opportunistic case finding in two general practices from May 2010 to January 2011.	Inclusion criteria: ever smokers aged 35–79 years. Exclusion criteria: prior diagnosis of COPD or asthma.	Active arm Stage 1: postal screening questionnaire. Stage 2: pre-BD spirometry in subjects with symptoms.	Opportunistic arm Stage 1: opportunistic screening questionnaire provided at routine primary care visits. Stage 2: pre-BD spirometry in subjects with symptoms.	Pre-BD FEV_1_/FVC<0.7 with FEV_1_<80% predicted, lack of reversibility (reversibility defined as increase in FEV_1_ of 200 ml and 15% from pre-BD FEV_1_) and presence of respiratory symptoms.	Active: 10/815 (1.2%). Opportunistic: 6/819 (0.7%). Difference in yield=0.5% (95% CI −0.5 to 1.5%).	Unclear whether outcome assessors were blinded. Poor spirometry attendance.
Konstantikaki 2011^25^	Non-randomised controlled trial: open spirometry programme versus case finding strategy in 24 semirural general practices from November 2008 to October 2009.	Inclusion criteria: >30 years. Exclusion criteria: history of respiratory tract infection in previous 4 weeks and inability to perform spirometry.	Open spirometry arm: public invitation through local advertisements offering free spirometry to people with chronic respiratory symptoms.	Case finding strategy: primary care physicians identified patients with a probable diagnosis of COPD in their daily practice and spirometry performed by research team.	History of exposure to noxious particles or gases, particularly smoking, compatible symptoms and post-BD FEV_1_/FVC<0.7.	Open spirometry: 76/1084 (7.0%). Case finding: 56/219 (25.6%). Difference in yield=18.6% (95% CI 12.6 to 24.6%).	No randomisation. Poor description of recruitment, selection criteria and spirometry.

Abbreviations: BD, bronchodilator; CI, confidence interval; COPD, chronic obstructive pulmonary disease; FEV_1_, forced expiratory volume in 1 s; FVC, forced vital capacity; RCT, randomised controlled trial.

a Subjects newly diagnosed with COPD.

b Eligible subjects.

**Table 2:** Studies evaluating spirometry, screening questionnaires and handheld flow meters

*Characteristic*		*Range (no. of studies)*		
		*Diagnostic spirometry* (*n*=30)	*Screening questionnaires* (*n*=13)	*Handheld flow meters* (*n*=5)
Study designs	RCT	1	1	0
	Cluster RCT	0	1	0
	Non-randomised trial	1	0	0
	Test accuracy study	7	8	4
	Single-arm study	21	3	1
Participants^a^	Screened	—	18,932	4,759
	Performed spirometry	63,087	8,845	568
	Diagnosed with COPD	10,428	1,996	346
Mean age (years)		47.9–65.3	52.3–65.3	52–65
Male (%)		19.6–100	38.1–69	37.7–99.7
Required smoking status	Current/ex-smokers	11	7	2
	Inc. never smokers	19	6	3
Required respiratory symptoms		5	0	0
Setting	General practice(s)	24	12	5
	Pharmacies	1	1	0
	Other	3	0	0
	Not reported	2	0	0
Number of centres		1–821	1–36	3–25
	Multicentre	24	12	5
	Single centre	2	1	0
	Not reported	4	0	0
Recruitment strategy	Active	13	6	1
	Opportunistic	14	4	3
	Active and opportunistic	1	2	1
	Not reported	2	1	0
Questionnaires	CDQ^1^	—	6	—
	LFQ	—	2	—
	Not named	—	5	—
Common items		—		—
	Age	—	11	—
	Smoking status	—	12	—
	Respiratory symptoms	—	13	—
	Allergies	—	7	—
				
*Handheld spirometers*				
Device	Piko-6	—	—	4
	COPD-6	—	—	1
Operator	Nurse	—	—	3
	GP	—	—	1
	Not reported	—	—	1
Use of bronchodilator	Pre-bronchodilator	—	—	3
	Post-bronchodilator	—	—	2
Test threshold	FEV_1_/FEV_6_<0.7	—	—	3
	FEV_1_/FEV_6_<0.75	—	—	1
	FEV_1_/FEV_6_<0.8	—	—	1
				
*Spirometry*				
Post-bronchodilator		15	10	5
Pre-bronchodilator		13	3	0
Not reported		2	0	0
Definition of airflow obstruction	Post-BD FEV_1_/FVC<0.7	12	9	3
	Pre-BD FEV_1_/FVC<0.7	9	2	0
	Post-BD FEV_1_/FVC<LLN	1	0	0
	Pre-BD FEV_1_/FVC<LLN	1	0	0
	Other	7	2	2
Symptoms in definition of COPD		4	3	0
Spirometry quality control	Yes	22	11	2
	No	4	2	1
	Unclear	4	0	2
				
*Range of results*				
New COPD cases/eligible subjects^a^		1.7–30.5%^19^	0.4–22.3%^8^	6–20%^3^
New COPD cases/no. screened		—	1.5–30.0%^12^	3–20%^5^
New COPD cases /no. assessed with spirometry		4.1–40.2%^30^	14.3–42.1%^13^	19–94%^5^
Severity of new cases (FEV_1_% predicted)^b^	⩾80%	11.5–86.7%	11.5–51.1%	33.3–64.3%
	50–80%	12.9–68.2%	42.8–87.5%	35.7–61.4%
	<50%	0–37.2%	5.3–37.2%	0–16.2%

Abbreviations: BD, bronchodilator; CDQ, COPD Diagnostic Questionnaire (also referred to as the Respiratory Health Screening Questionnaire and the International Primary Airways Group Questionnaire); COPD, chronic obstructive pulmonary disease; FEV_1_, forced expiratory volume in 1 s; FVC, forced vital capacity; LFQ, Lung Function Questionnaire; LLN, lower limit of normal; RCT, randomised controlled trial.

a A number of studies did not report the total eligible population.

b Restricted to studies that reported severity staging according to the Global Initiative for Obstructive Lung Disease (GOLD) strategy.^16^

## Notes

REJ and PA are principal investigators on the TargetCOPD trial, which evaluates the effectiveness of different approaches to case finding for COPD in primary care. TargetCOPD is funded by the National Institute for Health Research under its Programme Grants for Applied Research Programme (Grant Reference Number RP-PG-0109-10061).
