# Supplementary material for: Effectiveness of case finding strategies for COPD in primary care: a systematic review and meta-analysis
Source: NPJ Prim Care Respir Med. 2015 Aug 27;25:15056–. doi: 10.1038/npjpcrm.2015.56 (PMC4551096; doi:10.1038/npjpcrm.2015.56)
Supplement: Supplementary Tables [file npjpcrm201556-s1.doc]

# Supplementary tables and figures

Table S1 Search terms

| **Population** | **AND** | **Index test** |
| --- | --- | --- |
| Chronic obstructive pulmonary disease |  | Case finding |
| **OR** |  | **OR** |
| Chronic obstructive airways disease |  | Screening |
| **OR** |  | **OR** |
| Chronic obstructive lung disease |  | Early detection |
| **OR** |  | **OR** |
| COPD |  | Secondary prevention |
| **OR** |  | **OR** |
| COAD |  | Spirometry |
| **OR** |  | **OR** |
| Emphysema |  | Questionnaire |
| **OR** |  | **OR** |
| Chronic bronchitis |  | Peak flow |
| **OR** |  | **OR** |
| Airflow obstruction |  | Chest X-ray |
| **OR** |  | **OR** |
| Airflow limitation |  | Decision aid |
|  |  | **OR** |
|  |  | Algorithm |
|  |  | OR |
|  |  | Sensitivity |
|  |  | OR |
|  |  | Specificity |

**Table S2** Study characteristics

| **Study** | **Country** | **Study design** | **Setting** | **Recruitment method** | **Eligibility criteria** | **Screening & diagnostic test(s)** |
| --- | --- | --- | --- | --- | --- | --- |
| **Al Ghobain 2011**[**^1^**](#_ENREF_1) | Saudi Arabia | Single arm before-after study | 60 primary healthcare clinics | Invited patients routinely attending private primary healthcare clinics. Dates of recruitment not reported. | Inclusion criteria  Age ≥40 years &  current/ex-smoker of >5 years duration  Exclusion criteria  Known lung disease, upper respiratory tract infection or spirometry contraindications | Pre-/post-BD spirometry |
| **Bednarek 2008**[**^2^**](#_ENREF_2) | Poland | Single arm before-after study | Single primary care practice | Personalised letter from primary care physician. Dates of recruitment not reported. | Inclusion criteria  Age ≥40 years  Exclusion criteria  None | Pre-/post-BD spirometry |
| **Broekhuizen 2010**[**^3^**](#_ENREF_3) | Netherlands | Single arm before-after study | 73 general practitioners | Invited patients visiting their GP for persistent cough lasting ≥14 days during the winter period from January 2006-April 2009 | Inclusion criteria  Age >50 years & persistent cough lasting ≥14 days  Exclusion criteria  Known COPD or asthma, suspected pneumonia, severe psychiatric symptoms, or terminal illness | Extensive diagnostic work-up 90 days after presentation, including pre- /post-BD spirometry |
| **Buffels**  **2004**[**^4^**](#_ENREF_4) | Belgium | Cross-sectional  test accuracy study | 20 general practitioners | Invited patients routinely attending general practice over a 12 week period in 1999 | Inclusion criteria  Age 35-70 years  Exclusion criteria  Receiving bronchodilators and/or inhaled corticosteroids | Stage 1  Screening questionnaire  Stage 2  Pre-BD spirometry in all subjects with respiratory symptoms and 10% sample of asymptomatic subjects |
| **Bunker**  **2009** [**^5^**](#_ENREF_5) | Australia | RCT (case finding vs. usual care) | 4 general practices | Postal invitation with two reminder letters. Dates of recruitment not reported. | Inclusion criteria  Current/ex-smokers aged 40-80 years  Exclusion criteria  Known diagnosis of COPD, cognitive impairment, non-English speaking, <2 visits to practice in preceding year | Spirometry (unclear whether pre- or post-BD) |
| **Castillo**  **2009**[**^6^**](#_ENREF_6) | Spain | Single arm before-after study | 13 community pharmacies | Invited subjects visiting participating pharmacies in April/May 2007 | Inclusion criteria  Age >40 years  Exclusion criteria  History of lung disease or use of inhalers | Stage 1  Screening questionnaire  Stage 2  Pre-BD spirometry for those with ≥3 positive answers |
| **Clotet**  **2004**[**^7^**](#_ENREF_7) | Spain | Single arm before-after study | NR | NR | Inclusion criteria  Age 40-76 years & active smoker for ≥10 years with no or only mild respiratory symptoms  Exclusion criteria  Previous diagnosis of COPD, asthma, bronchiectasis, cystic fibrosis, tuberculosis, chronic bronchitis, restrictive pulmonary disease or receiving bronchodilators | Pre-BD spirometry |
| **DeJong**  **2004**[**^8^**](#_ENREF_8) | US | Single arm before-after study | Range of settings – unclear | Direct mailing to 1500 individuals who were coded as being a current/ex- smoker within a large primary care clinic system.  Advertised via article in the local newspaper and posters placed in 6 physician offices and 1 hospital. (Dates not reported) | Inclusion criteria  Age 40-60 years (although no subjects were turned away)  Exclusion criteria  None | Pre-BD spirometry |
| **Dirven**  **2013a**[**^9^**](#_ENREF_9) | Netherlands | Cluster RCT (patient vs. practice-managed screening approach) | 16 general practices in four cities | The COPD Diagnostic Questionnaire (referred to as the “Respiratory Health Screening Questionnaire”) was posted to all eligible patients registered at participating practices from May to September 2012 | Inclusion criteria  Age 40-70 years  Exclusion criteria  Previous diagnosis of asthma, COPD or significant lung disease such as lung cancer, pneumoconiosis, tuberculosis, bronchiectasis and pneumonectomy. Also excluded patients using oxygen supplementation and with impaired mobility. | Stage 1  In the “patient-managed” arm subjects were asked to calculate their risk of COPD using a screening questionnaire (COPD Diagnostic Questionnaire) and advised to consult their GP if their score was >19.5. In the “practice-managed” arm the scoring was performed by the practice and subjects with a score >19.5 were invited for spirometry.  Stage 2:  Post-BD spirometry for subjects with a score >19.5 |
| **Dirven**  **2013b**[**^10^**](#_ENREF_10) | Netherlands | Single arm before-after study | 10 general practices in two cities (five low socioeconomic status (SES) practices and five moderate-to-high SES) | The COPD Diagnostic Questionnaire (referred to as the “Respiratory Health Screening Questionnaire”) was posted to all eligible patients registered at participating practices from May-July 2012. | Inclusion criteria  Age 40-70 years  Exclusion criteria  As above (Dirven 2013a) | Stage 1:  Screening questionnaire (COPD Diagnostic Questionnaire; score completed by practice assistant)  Stage 2:  Post-BD spirometry for subjects with a score ≥19.5 |
| **Duong-Quy 2009**[**^11^**](#_ENREF_11) | Vietnam | Cross-sectional test accuracy study | 12 primary care medical centres in one city | Broadcast an advertisement on the local television daily for one week. A recruitment company was used to help with recruiting participants (details not reported). Eligible subjects expressing an interest in participating were advised to attend one of the twelve primary care centres from January 2007- February 2008. | Inclusion criteria  Current/ex-smokers aged >40 years with >10 pack-years  Exclusion criteria  Previously diagnosed respiratory disease (asthma, COPD and tuberculosis) | Stage 1  Pre-BD handheld flow meter (Piko-6®)  Stage 2  Full medical assessment including clinical examination, pulmonary radiology, ECG, and post-BD spirometry for subjects with FEV_1_/FEV_6_<0.7 and a sample of subjects with FEV_1_/FEV_6_≥0.7 |
| **Freeman**  **2005**[**^12^**](#_ENREF_12) | UK | Cross-sectional test accuracy study | One general practice | Postal invitation from October 1997-April 2002 | Inclusion criteria  Current/ex-smokers aged ≥40 years & had either received respiratory medications in the preceding two years or history of asthma | Stage 1  Screening questions  Stage 2  Pre-/ post-BD spirometry on all subjects |
| **Frith**  **2011**[**^13^**](#_ENREF_13) | Australia | Cross-sectional test accuracy study | Four primary care practices | Recruited during routine practice visits, invitation to study days, and via local newspaper advertisement between August-December 2006 | Inclusion criteria  Current/ex-smokers aged ≥50 years with no prior diagnosis of obstructive lung disease (COPD, emphysema, chronic bronchitis, asthma) & no treatment for obstructive lung disease in past 12 months  Exclusion criteria  Refusal or inability to give consent, pre-existing non-obstructive lung disease, symptoms suggestive of unstable heart disease, and spirometry contraindications | Stage 1:  Pre-BD Piko-6® & screening questionnaire (COPD Diagnostic Questionnaire)  Stage 2:  Pre-/ post-BD spirometry on all subjects |
| **Fuller**  **2012**[**^14^**](#_ENREF_14) | US | Single arm before-after study | Four community pharmacies and offsite screening events. Each pharmacy was an established patient care centre which had a clinical pharmacist and provided services such as immunisation, medication therapy management and smoking cessation counselling | Screening was offered to any interested subjects fulfilling the eligibility criteria. Participation was encouraged by in-store advertising, brochures, prescription bag ties and fliers.  Recruitment took place from March-June 2010 | Inclusion criteria  Age ≥35 years  Exclusion criteria  History of lung cancer, pregnancy at time of screening, previous diagnosis of COPD, lung surgery or resection, recent abdominal or thoracic surgery, respiratory infection within the previous 3 weeks, uncontrolled hypertension (>160/90 mm Hg),and inability to produce three acceptable tracings during spirometry | Pre-BD spirometry |
| **Geijer**  **2005**[**^15^**](#_ENREF_15) | Netherlands | Single arm before-after study | Primary care (otherwise unspecified) | Mailed invitation ascertaining smoking status, only inviting current smokers in 1998 with follow-up measurements in 2003 | Inclusion criteria  Males aged 40-65 years who had smoked ≥1 cigarette per day during previous 12 months  Exclusion criteria  Prior history of lung disease, ex-/non-/pipe/cigar smokers | Pre-/post-BD spirometry (post-BD spirometry only performed if pre-BD FEV_1_ % predicted<85%) |
| **Hanania**  **2010**[**^16^**](#_ENREF_16) | US | Cross-sectional test accuracy study | Two family physician group offices | Invited patients visiting the practices from March-May 2008 | Inclusion criteria  Age ≥40 years  Exclusion criteria:  None | Stage 1  Screening questionnaire (Lung Function Questionnaire)  Stage 2  Pre-BD spirometry in all subjects |
| **Haroon**  **2013**[**^17^**](#_ENREF_17) | UK | RCT (targeted vs. opportunistic case finding) | Two general practices | Postal invitation (“targeted”) vs. invitation to patients routinely attending primary care (“opportunistic”) from May 2010-January 2011 | Inclusion criteria  Current/ex-smokers aged 35-79 years  Exclusion criteria  Prior diagnosis of COPD or asthma | Stage 1  Screening questionnaire  Stage 2  Pre-BD spirometry in subjects with symptoms |
| **Kaufmann 2009**[**^18^**](#_ENREF_18) | Austria | Single arm before-after study | Three general practices | Invited patients who attended participating practices from May-June 2005 | Inclusion criteria  NR  Exclusion criteria  Known lung or psychiatric disease | Stage 1  Pre-BD handheld flow meter (Piko-6®)  Stage 2  Post-BD spirometry |
| **Kimura**  **2011**[**^19^**](#_ENREF_19) | Japan | Repeated single arm before-after studies (reported two separate studies) | Community health centre | Personalised postal invitation from the local government to the general population from 2004-2007. COPD was described in a newsletter and a lecture was delivered by one of the authors before the study, emphasizing the prevention of severe life-threatening disease | Inclusion criteria  Age >40 years (study 1). Inclusion criteria not specified for study 2  Exclusion criteria  Illiteracy | Pre-BD spirometry |
| **Kögler**  **2010**[**^20^**](#_ENREF_20) | Germany | Single arm before-after study | 684 urban and rural primary care practices and 137 respiratory physicians | Invited consecutive patients routinely attending primary care (dates not reported) | Inclusion criteria  Age ≥40 years with no prior diagnosis of lung disease.  Subjects were then selected either if they had ever smoked or regularly suffered from cough and/or breathlessness. | Assessment by a pulmonologist, including spirometry (unclear whether pre- or post-BD) |
| **Konstantikaki 2011**[**^21^**](#_ENREF_21) | Greece | Non-randomised controlled trial | 24 semirural primary care practices | Public invitation with local advertisement offering free spirometry to individuals with chronic respiratory symptoms (open spirometry programme) from November 2008-October 2009.  Primary care physicians identified patients with a probable diagnosis of COPD in their daily practice (case finding strategy). | Inclusion criteria  >30 years  Exclusion criteria  History of respiratory tract infection in previous four weeks and inability to perform spirometry | Post-BD spirometry |
| **Kotz**  **2008**[**^22^**](#_ENREF_22) | Netherlands | Cross-sectional test accuracy study | General population and primary care practices | Advertisements in a local newspaper, flyers, posters, mailings to households and invitation during primary care consultations from  January 2005-December 2006 | Inclusion criteria  Current smokers aged 40-70 years with ≥10 pack years & motivated to stop smoking, able to read and speak Dutch & reporting a respiratory symptom (cough, phlegm or dyspnoea)  Exclusion criteria:  Prior respiratory diagnosis, spirometry in previous 12 months or contraindications to smoking cessation therapy | Stage 1  Questionnaire (COPD Diagnostic Questionnaire)  Stage 2  Pre-/post-BD spirometry in all participants |
| **Laniado-Laborin**  **2011**[**^23^**](#_ENREF_23) | Mexico | Single arm before-after study | Primary care practices in 27 cities from March-October 2008 | Received phone call from family physician to schedule an assessment from March-October 2008 | Inclusion criteria  Age ≥40 years  Exclusion criteria  None | Stage 1  Screening questionnaire  Stage 2  Subjects with risk factors or symptoms proceeded to post-BD spirometry |
| **Leuppi**  **2010**[**^24^**](#_ENREF_24) | Switzerland | Single arm before-after study | Primary care practices with 440 GPs | Invited patients attending primary care (dates not reported) | Inclusion criteria  Current smokers aged ≥40 years  Exclusion criteria  None | Pre-BD spirometry |
| **Løkke**  **2012**[**^25^**](#_ENREF_25) | Denmark | Single arm before-after study | Primary care practices with 241 GPs | Invited consecutive patients attending practices during March-August 2010 | Inclusion criteria  Age ≥35 years & current/ex-smoker or relevant occupational exposure & ≥1 respiratory symptom (dyspnoea, cough, wheeze, sputum and/or recurrent chest infections)  Exclusion criteria  Unable to perform spirometry or previous diagnosis of chronic respiratory disease | Pre-BD spirometry with bronchodilator reversibility for patients with airway obstruction and corticosteroid reversibility test for subjects with an increase in FEV_1_ between 200-500mL |
| **Mintz**  **2011**[**^26^**](#_ENREF_26) | US | Cross-sectional test accuracy study | 36 primary care centres | NR.  Study took place from 18^th^ February to 29^th^ May 2009. | Inclusion criteria  Current/ex-smokers aged ≥30 years old with ≥10 pack years  Exclusion criteria  Regular use of respiratory medication within 4 weeks of study, known diagnosis of substantial lung conditions with regular use of respiratory medication. | Stage 1  Screening questionnaire (Lung Function Questionnaire)  Stage 2  Pre-/post-BD spirometry on subjects with LFQ ≤18 & first two subjects with LFQ ≥18 at each site |
| **Price**  **2006**[**^27^**](#_ENREF_27) | UK & US | Cross-sectional test accuracy study | Two primary care practices | Postal invitation (dates not reported) | Inclusion criteria  Current/ex-smokers aged ≥40 years  Exclusion criteria  Refusal to consent, history of non-obstructive lung disease, use of respiratory medications in previous year, and acute symptoms of unstable heart disease | Stage 1  Screening questionnaire (COPD Diagnostic Questionnaire)  Stage 2  Pre-/post-BD spirometry on all subjects |
| **de Queiroz**  **2012**[**^28^**](#_ENREF_28) | Brazil | Single arm before-after study | Three primary healthcare clinics | Invited individuals routinely attending primary healthcare clinics between May-September 2011 | Inclusion criteria  Age ≥40 years & ≥20 pack-years smoking or ≥80 hour-year history of exposure to biomass smoke  Exclusion criteria  Acute respiratory symptoms, previous history of bronchial asthma, allergic rhinitis, chronic lung diseases other than COPD, extra-pulmonary disease potentially affecting lung function and not meeting the criteria for spirometry | Pre-/post-BD spirometry |
| **Sandelowsky 2011**[**^29^**](#_ENREF_29) | Sweden | Single arm before-after study | Primary healthcare centre and urgent primary care unit | Patients meeting the inclusion criteria were telephoned from January-March 2005. Telephone conversations were followed by formal written invitations. | Inclusion criteria  Current/ex-smokers aged 40-75 years & visited a primary healthcare centre or urgent primary care unit & diagnosed with acute respiratory infection (confirmed with a review of the medical records).  Exclusion criteria  Poor knowledge of Swedish, severe cardiac, psychiatric or multi-organ disease, prior history of lung disease (except asthma), or on beta-blockers | Pre-/post-BD spirometry |
| **Sichletidis 2011**[**^30^**](#_ENREF_30) | Greece | Cross-sectional test accuracy study | 25 general practices | Invited first 50 patients meeting the inclusion criteria who attended each participating GP from March-May 2009 | Inclusion criteria  Age >40 years  Exclusion criteria  Confirmed diagnosis of lung disease, thoracic surgery in previous six months, acute respiratory infection, uncontrolled cardiac disease, or could not perform acceptable spirometry | Stage 1  Screening questionnaire (International Primary Airways Group [IPAG] questionnaire, also known as COPD Diagnostic Questionnaire)  Stage 2  Post-BD Piko-6® (using 400µg salbutamol)  Stage 3  Pre-/post-BD spirometry |
| **Stratelis**  **2004**[**^31^**](#_ENREF_31) | Sweden | Single arm before-after study | Primary care centres (number of centres not reported) | Placards advertising the study displayed in each healthcare centre and advertisement in local newspaper (dates not reported) | Inclusion criteria  Current smokers aged 40-55 years (or had stopped smoking within 3 months of the study)  Exclusion criteria  None | Pre-BD spirometry |
| **Takahashi 2003**[**^32^**](#_ENREF_32) | Japan | Single arm before-after study | 56 primary care facilities (23 hospitals and 33 general practices) | Not reported | Inclusion criteria  Considered to be high risk for COPD (not otherwise defined)  Exclusion criteria  Established diagnosis of COPD, asthma or other chronic respiratory disease | Pre-BD spirometry |
| **Takemura 2005**[**^33^**](#_ENREF_33) | Japan | Single arm before-after study | Health screening clinic | Invited patients attending a medical check-up from 1997-2001 | Inclusion criteria  Age >30 years  Exclusion criteria  Established diagnosis of chronic respiratory disease, abnormalities on chest X-ray and unknown smoking status | Pre-BD spirometry |
| **Thorn**  **2012**[**^34^**](#_ENREF_34) | Sweden | Cross-sectional test accuracy study | 21 primary healthcare centres | Invited patients attending participating primary healthcare centres over a five month period (dates not reported) | Inclusion criteria  Current/ex-smokers aged 45-85 years with ≥15 pack years  Exclusion criteria  None | Stage 1  Pre-BD handheld spirometer (COPD-6®)  Stage 2  Pre-/post-BD spirometry |
| **Ulrik**  **2011**[**^35^**](#_ENREF_35) | Denmark | Single arm before-after study | Primary care practices involving 335 GPs | Invited consecutive patients routinely attending primary care (dates not reported) | Inclusion criteria  Age ≥35 years old  & current/ex- smoker or relevant occupational exposure &  ≥1 respiratory symptom (dyspnoea, cough, wheeze, sputum or recurrent chest infections)  Exclusion criteria  Unable to perform spirometry or previous diagnosis of chronic respiratory disease | Pre-BD spirometry |
| **van Schayck 2002**[**^36^**](#_ENREF_36) | Netherlands | Single arm before-after study | Two semirural primary care practices | Randomly invited patients routinely attending primary care (dates not reported) | Inclusion criteria  Current smokers aged 35-70 years  Exclusion criteria  Use of respiratory medication | Pre-BD spirometry |
| **Vandevoorde 2007**[**^37^**](#_ENREF_37) | Belgium | Single arm before-after study | Six primary care practices, involving eight GPs | Invited patients routinely attending primary care over a two month period (dates not reported) | Inclusion criteria  Current smokers aged 40-70 years with ≥15 pack-years  Exclusion criteria  History of asthma or COPD | Pre-BD spirometry |
| **Vrijhoef**  **2003**[**^38^**](#_ENREF_38) | Netherlands | Single-arm before-after study | Eight primary care practices | Invited patients attending primary care for reasons unrelated to respiratory disease from September 1998-July 1999 | Inclusion criteria  Current/ex-smokers aged 40-70 years  Exclusion criteria  Receiving respiratory medication, known diagnosis of asthma, COPD or chronic bronchitis, or significant co-morbidity | Stage 1  Pre-/ post-BD spirometry performed by medical undergraduates  Stage 2:  Pre-/post-BD spirometry performed by specialist respiratory nurse |

ATS=American Thoracic Society, BD=bronchodilator, ERS=European Respiratory Society, NR=not reported, RCT=randomised controlled trial

**Table S3** Quality assessment of randomised controlled trials

|  | **Bunker 2009** | **Haroon 2013** | **Dirven 2013a** |
| --- | --- | --- | --- |
| Adequate description of method of randomisation | No | Yes | Yes |
| Adequate description of method of allocation concealment | No | No | No |
| Comparison groups similar except for intervention received | Unclear | Yes | Unclear |
| Participants blinded to intervention arm | Yes | Yes | Yes |
| Outcome assessors blinded to intervention arm | No | No | Unclear |
| Outcome data completeness described for each outcome, including attrition and exclusions from the analysis | No | Yes | Yes |
| Evidence of selective outcome reporting | No | No | No |
| Other sources of bias | Inadequate outcome ascertainment in control group | Poor spirometry attendance in both arms | Did not report number of spirometry procedures that were of acceptable quality |

**Table S4**a Quality assessment of studies evaluating spirometry

|  | **Al Ghobain 2011** | **Bednarek 2008** | **Broekhuizen 2010** | **Bunker 2009** | **Clotet 2004** | **DeJong 2004** | **Fuller 2012** | **Geijer 2005** | **Kimura 2011** | **Kögler 2010** | **Konstantikaki 2011** | **Leuppi 2010** | **Løkke 2012** | **de Queiroz 2012** | **Sandelowsky 2011** | **Stratelis 2004** | **Takahashi 2003** | **Takemura 2005** | **Ulrik 2011** | **Van Shayck 2002** | **Vandevoorde 2007** | **Vrijhoef 2003** |
| --- | --- | --- | --- | --- | --- | --- | --- | --- | --- | --- | --- | --- | --- | --- | --- | --- | --- | --- | --- | --- | --- | --- |
| Clear description of recruitment | Y | Y | Y | Y | N | Y | Y | Y | Y | Y | N | N | Y | Y | Y | Y | N | N | Y | Y | Y | Y |
| Clear description of selection criteria | Y | Y | Y | Y | Y | N | Y | Y | N | Y | N | Y | Y | Y | Y | Y | N | Y | Y | Y | Y | Y |
| Clear description of participants | Y | Y | Y | N | N | Y | Y | Y | N | Y | Y | N | Y | N | Y | Y | Y | Y | Y | N | Y | Y |
| Representative spectrum of patients | Y | Y | Y | U | U | Y | Y | N | U | Y | N | Y | Y | U | Y | N | Y | Y | Y | U | Y | Y |
| Clear description of withdrawals | N | Y | U | N | N | N | N | N | N | Y | Y | N | N | Y | Y | N | N | N | N | Y | U | Y |
| Participant flow diagram | N | N | N | Y | Y | N | Y | N | Y | N | Y | N | N | Y | Y | N | N | N | N | N | N | Y |
| Spirometry quality control | Y | Y | U | Y | N | N | Y | Y | Y | U | Y | Y | U | N | U | Y | Y | Y | N | Y | Y | Y |
| Standard diagnostic criteria | Y | Y | Y | Y | Y | Y | Y | Y | N | Y | Y | Y | Y | Y | Y | Y | Y | Y | Y | Y | Y | N |
| Intervention described in sufficient detail to permit its replication | Y | Y | Y | Y | N | N | Y | Y | Y | Y | N | Y | Y | Y | Y | Y | Y | Y | N | Y | Y | Y |
| Clinical data available representative of routine practice | Y | Y | Y | Y | Y | Y | Y | Y | Y | Y | Y | Y | Y | Y | Y | Y | Y | Y | Y | Y | Y | Y |
| Uninterruptable, indeterminate or intermediate results reported | Y | Y | N | N | N | N | Y | Y | Y | N | Y | Y | N | Y | Y | N | Y | N | N | Y | Y | N |

Y=yes, N=no, U=unclear

Table S4b Quality assessment of studies evaluating screening questionnaires and/or handheld flow meters

|  | **Buffels 2004** | **Castillo 2009** | **Dirven 2013a** | **Dirven 2013b** | **Duong-Quy 2009** | **Freeman 2005** | **Frith 2011** | **Hanania 2010** | **Haroon 2013** | **Kaufmann 2009** | **Kotz 2008** | **Laniado-Laborin 2011** | **Mintz 2011** | **Price 2006** | **Sichletidis 2011** | **Thorn 2012** |
| --- | --- | --- | --- | --- | --- | --- | --- | --- | --- | --- | --- | --- | --- | --- | --- | --- |
| Clear description of recruitment | Y | Y | Y | Y | Y | Y | Y | Y | Y | Y | Y | U | N | Y | Y | Y |
| Clear description of selection criteria | Y | Y | Y | Y | Y | Y | Y | Y | Y | Y | Y | Y | Y | Y | Y | Y |
| Clear description of participants | N | Y | Y | N | Y | Y | Y | Y | Y | Y | Y | Y | Y | Y | Y | Y |
| Representative spectrum of patients | U | Y | Y | U | N | Y | Y | U | Y | Y | Y | Y | Y | Y | Y | Y |
| Clear description of withdrawals | Y | Y | N | Y | Y | N | N | U | N | Y | Y | Y | Y | Y | Y | N |
| Participant flow diagram | Y | Y | N | N | N | N | Y | N | Y | N | Y | N | Y | Y | N | N |
| Spirometry quality control | Y | Y | N | N | N | Y | Y | Y | Y | U | Y | Y | Y | Y | Y | U |
| Standard diagnostic criteria | Y | Y | Y | Y | Y | Y | Y | Y | Y | U | Y | Y | Y | Y | Y | Y |
| Quality diagnostic spirometry as reference standard | Y | Y | Y | U | Y | Y | Y | Y | Y | U | Y | Y | Y | Y | Y | Y |
| Spirometry performed within 6 months of index test | Y | Y | U | U | U | Y | Y | Y | Y | U | U | Y | Y | Y | U | Y |
| All or random selection of participants with positive screening test result underwent spirometry | Y | Y | N | Y | N | Y | Y | Y | N | Y | Y | Y | N | Y | Y | Y |
| Intervention described in sufficient detail to permit its replication | Y | Y | Y | Y | Y | Y | Y | Y | Y | N | Y | Y | Y | Y | Y | Y |
| Clinical data available representative of routine practice | Y | Y | Y | Y | Y | Y | Y | Y | Y | Y | Y | Y | Y | Y | Y | Y |
| Uninterpretable, indeterminate or intermediate results reported | N | Y | N | N | N | N | Y | N | N | U | Y | Y | Y | Y | Y | N |

Y=yes, N=no, U=unclear

**Table S5 Results: studies evaluating** spirometry

| **Study** | **Population** | **Spirometry** | **Spirometry** | **Definition of COPD** | **New cases of COPD^1^** |
| --- | --- | --- | --- | --- | --- |
| **Al Ghobain**  **2011**[**^1^**](#_ENREF_1) | Eligible: 1380  Invited: 1380  Subjects with acceptable spirometry:  Mean age: 47.9  Male: 89.6%  Smoking status  Current: 82.6%  Former: 17.4% | Device: NR  Bronchodilator: 200µg salbutamol  Operator: Physicians trained in the research methods  Standard: ATS standards  Quality control: Two investigators independently assessed the quality of spirometry tests according to ATS criteria. | Total: 1380  Acceptable: 501 | Post-BD FEV_1_/FVC<0.7 with no history of asthma or atopy, and with a smoking history of ≥5 years | 71 cases  FEV_1_ % predicted  >70%: 0  50-70%: 40 (56.3%)  <50%: 31 (43.6%) |
| **Bednarek**  **2008**[**^2^**](#_ENREF_2) | Eligible: 2250  Invited: NR  Subjects with acceptable spirometry  Mean age: 56.7  Male: 39.0%  Smoking status  Current: 29.6%  Former: 24.9%  Never: 45.5% | Device: EasyOne Diagnostic (ndd Medical)  Bronchodilator: 200µg salbutamol  Operator: Primary care nurses who had attended two four hour training sessions in a reference lung function laboratory  Standard: ATS standards  Quality control: Spirometry tests were sent to one of the authors for quality assessment, reviewed according to ATS criteria | Total: 1986  Acceptable: 1960 | Post-BD FEV_1_/FVC<5^th^ percentile of normal (using ECCS predicted values) & compatible clinical presentation (excluding asthma based on case notes review) | 149 cases  FEV_1_ % predicted  >70%: 56 (30.6%)  50-69%: 94 (51.4%)  35-49%: 28 (15.3%)  <35%: 5 (2.7%) |
| **Broekhuizen 2010**[**^3^**](#_ENREF_3) | Eligible: NR  Invited: NR  Subjects analysed:  Mean age: 63  Male: 45%  Smoking status  Current/ex: 74%  Never: 26% | Device: NR  Bronchodilator: 400µg salbutamol  Operator: Lung function technicians  Standard: NR  Quality control: Tests performed in a secondary care lung function laboratory | Total: 353  Acceptable: NR | Consensus panel diagnosis with two physicians. Diagnosis based on recurrent respiratory symptoms & post-BD FEV_1_/FVC<0.7 & clinical judgement | 102 cases  FEV_1_ % predicted  >80%: 71 (69.7%)  50-80%: 29 (28.4%)  30-50%: 2 (2.0%)  <30%: 0 |
| **Bunker**  **2009**[**^5^**](#_ENREF_5) | Eligible: 400  Invited: NR  Subjects who performed spirometry:  Mean age: 62.4*  Male: 57.0%  Smoking status  Current: 32.9%  Former: 67.1% | Device: NR  Bronchodilator: NR  Operator: practice nurses who had attended a two hour training session on performance and interpretation of spirometry  Standard: GOLD strategy  Quality control: 25% of spirometry results were reviewed by a member of the study team. | Total: 79  Acceptable: NR | FEV_1_/FVC<70% (unclear whether pre- or post-BD) | 10 cases  FEV_1_ % predicted  >80%: 5 (31.3%)  50-80%: 9 (56.3%)  30-50%: 2 (12.5%)  <30%: 0  NB. FEV1 % predicted includes cases that were later deemed not to have COPD. |
| **Clotet**  **2004**[**^7^**](#_ENREF_7) | Eligible: 177  Invited: 177  Subjects with spirometry:  Mean age: 54  Male: 80%  Smoking status  Current: 100% | Device: DATOSPIR 100 spirometer  Bronchodilator: None  Operator: NR  Standard: Spanish Society of Pulmonology and Thoracic Surgery (SEPAR)  Quality control: NR | Total: 155  Acceptable: NR | Pre-BD FEV_1_<80% predicted & FEV/FVC<70% | 36 cases  FEV_1_ % predicted  NR |
| **DeJong**  **2004**[**^8^**](#_ENREF_8) | Eligible: NR  Invited: NR  Subjects who performed spirometry:  Mean age: 55  Male: 49%  Smoking status  Current: 49%  Former: 37%  Never: 14% | Spirometer: Jones handheld spirometer  Bronchodilator: NR  Operator: Trained respiratory therapist  Standard: NR  Quality control: NR | Total: 243  Acceptable: NR | Pre-BD FEV1/FVC<0.7 | 43 cases  FEV1 % predicted:  >80%: 15 (34.9%)  50-80%: 28 (65.1%)  30-50%: 0  <30%: 0 |
| **Freeman**  **2005**[**^12^**](#_ENREF_12) | Eligible: 1195  Invited: 1195  Consented: 624  Subjects who performed spirometry  Mean age: 61.7  Male: 52%  Smoking status  Current: 54.1%  Former: 45.9% | Spirometer: Micro-Med handheld spirometer with Spida software  Bronchodilator: 5mg salbutamol for those with prior respiratory medication/history of asthma/FEV_1_<80% predicted  Operator: Trained respiratory nurse  Standard: ATS standards. Minimum of 3 tests or until results within 5%.  Quality control: All spirometry results were reviewed by a physician to ensure compliance with ATS standards | Total: 369  Acceptable: NR | Post-BD FEV_1_/FVC<0.7 and lack of reversibility (reversibility defined as increase in FEV_1_ ≥200mL and 15% from pre-BD FEV_1_  (not clear if all were post-BD) | 62 cases  FEV1 % predicted  NR |
| **Frith**  **2011**[**^13^**](#_ENREF_13) | Eligible: 233  Invited: 237  Subjects with acceptable spirometry:  Mean age: 61  Male: 69%  Smoking status  Current: 45%  Former: 55%  Never: <1% | Device: EasyOne spirometer (ndd Medical)  Bronchodilator: 360mcg salbutamol  Operator: trained operators using ATS/ERS guidelines  Standard: ATS/ERS standards. At least 3 adequate baseline and post-BD FVC manoeuvres performed.  Quality control: spirometry quality monitored by a respiratory physiologist blinded to the questionnaire and Piko-6® results | Total: 233  Acceptable: 204 | Post-BD FEV_1_/FVC<0.7 | 57 cases  FEV_1_ % predicted  >80%: 19 (33.3%)  50-80%: 35 (61.4%)  30-50%: 3 (5.3%)  <30%: 0 |
| **Fuller**  **2012**[**^14^**](#_ENREF_14) | Eligible: NR  Invited: NR  Subjects with acceptable spirometry:  Mean age: 55  Male: 40%  Smoking status  Current: 12%  Former: 34%  Never: 54% | Device: EasyOne spirometer (ndd Medical)  Bronchodilator: None  Operator: 3 pharmacy residents and 5 clinical pharmacists who had participated in 16 hours of training from a pulmonary physician experienced in lung function testing.  Standard: ATS standards  Quality control: All spirometry results were reviewed independently by two study pulmonologists. | Total: 185  Acceptable: 174 | Pre-BD FEV_1_/FVC<LLN (using NHANES III) reference equations | 16 cases  FEV_1_ % predicted  NR |
| **Geijer**  **2005**[**^15^**](#_ENREF_15) | Eligible: 918  Invited: NR  Subjects with acceptable spirometry:  Mean age: 50  Male: 100%  Smoking status  Current: 100% | Device: Vitalograph 2170 with Spirotrack software  Bronchodilator: 250µg terbutaline  Operator: Trained nurse practitioner  Standard: Each subject had to perform at least three acceptable FVC manoeuvres according to ATS standards.  Quality control: Two investigators independently assessed quality of spirometry curves according to ATS criteria. Where there was disagreement a final assessment was made by a lung physiologist. | Total: 805  Acceptable: 702 | FEV_1_/FVC<0.7 (unclear whether pre- or post-BD) | 210 cases  FEV_1_ % predicted  ≥80%: 182 (86.7%)  50-80%: 27 (12.9%)  30-49%: 1 (0.5%)  <30%: 0 |
| **Hanania**  **2010**[**^16^**](#_ENREF_16) | Eligible: NR  Invited: NR  Subjects with spirometry:  Mean age: NR  Male: 38.1%  Smoking status  NR | Device: EasyOne spirometers (ndd Medical Technologies)  Bronchodilator: None  Operator: NR  Standard: NR  Quality control: Investigators rated spirometry quality based on reliability and reproducibility and only included traces that were considered reliable. | Total: 937  Acceptable: NR | Pre-BD FEV_1_/FVC<0.7 | 156 cases  FEV_1_ % predicted  ≥80%: 17 (11.5%)  50-80%: 76 (51.4%)  30-50%: 44 (29.7%)  <30%: 11 (7.4%)  NB. Reported numbers do not summate to 156. |
| **Kimura**  **2011**[**^19^**](#_ENREF_19)  **(Study 1)** | Eligible: 839  Invited: NR  Eligible subjects:  Mean age: 63.3  Male: 33.7%  Smoking status  NR | Device: Chestgraph Jr. H1-101 (Chest Co.,  Japan)  Bronchodilator: None  Operator: trained examiners  Standard: ATS standards  Quality control: All spirometry data were checked by two study authors. Only tests with at least two manoeuvres with FEV_1_ within 0.2L were included in the analysis. | Total: 814  Acceptable: 630 | Pre-BD FEV_1_/FVC<0.7 | 59 cases  FEV_1_% predicted  ≥80%: 29 (49.2%)  50-80%: 27 (45.8%)  30-50%: 3 (5.1%)  <30%: 0 |
| **Kimura**  **2011**[**^19^**](#_ENREF_19)  **(Study 2)** | Eligible:1300  Invited:363  Mean age: NR  Male: NR  Smoking status  NR | As above | Total: 363  Acceptable: 254 | Pre-BD FEV_1_/FVC<0.7 | 22 cases  FEV_1_% predicted  ≥80%: 6 (27.3%)  50-80%: 15 (68.2%)  30-50%: 1 (4.5%)  <30%: 0 |
| **Kögler**  **2010**[**^20^**](#_ENREF_20) | Eligible: NR  Invited: 2276  Subjects with complete data:  Mean age: NR  Male: 62.2%*  Smoking status  Current/ex: 100% | Device: NR  Bronchodilator: NR  Operator: Pulmonologist  Standard: NR  Quality control: NR | Total: 1282  Acceptable: NR | Physician diagnosis (physicians were advised to follow the GOLD strategy) | 516 cases  FEV_1_% predicted:  ≥80%: 137 (12.6%)  50-80%: 290 (26.7%)  <50%: 89 (8.2%) |
| **Konstantikaki 2011**[**^21^**](#_ENREF_21)  **- Open spirometry programme** | Eligible: NR  Invited: NR  Subjects with acceptable spirometry:  Mean age: 62.5  Male: 64%  Smoking status  Current: 36.2%  Former: 27.1%  Never: 36.7% | Device: dry spirometer (Koko Legend, Ferraris, UK)  Bronchodilator: 400 µg salbutamol  Standard: ATS standards  Operator: Physicians who had undergone training by two chest physicians  Quality control: three experienced chest physicians evaluated the quality of all spirometry readings | Total:1084  Acceptable: 905 | History of exposure to noxious particles or gases, particularly smoking, compatible symptoms, and post-BD FEV_1_/FVC<0.7 | 76 cases  FEV_1_% predicted:  ≥80%: 34 (44.7%)  50-80%: 32 (42.1%)  30-50%: 8 (10.5%)  <30%: 2 (2.6%) |
| **Konstantikaki 2011**[**^21^**](#_ENREF_21)  **- Case-finding programme** | Eligible: NR  Invited: NR  Mean age: 63.5  Male: 19.6%  Smoking status  Current: 68.7%  Former: 22.9%  Never: 8.5% | As above | Total: 219  Acceptable: 201 | As above | 56 cases  FEV_1_% predicted  ≥80%: 18 (32.1%)  50-80%: 32 (57.1%)  30-50%: 4 (7.1%)  <30%: 2 (3.6%) |
| **Kotz**  **2008**[**^22^**](#_ENREF_22) | Eligible: 1052  Invited: 1052  Subject who performed spirometry:  Mean age: 52.3  Male: 58.7%  Smoking status  Current: 100% | Device: Vitalograph 2120  Bronchodilator: 500µg terbutaline  Operator: Two qualified research assistants under the supervision of a pulmonologist  Standard: ATS/ERS standards  Quality control: All spirometry results were validated by a pulmonologist and specialised lung function laboratory assistant not involved in the trial- both were blinded to the questionnaire scores | Total: 826  Acceptable: 716 | Post-BD FEV_1_/FVC<0.7 | 278 cases  FEV_1_ % predicted  ≥80%: 142 (51.1%)  50-80%: 119 (42.8%)  <50%: 17 (6.1%) |
| **Leuppi**  **2010**[**^24^**](#_ENREF_24) | Eligible: 26,400  Invited: NR  Subjects aged ≥40 years who performed spirometry:  Mean age: 51.6  Male: 58.6%  Smoking status  Current: 100% | Device: EasyOne spirometer (ndd Medical Technologies)  Bronchodilator: None  Operator: GPs and practice nurses provided with 1-2 hours training  Standard: ATS standards  Quality control: spirometry quality assessed automatically by the spirometer. Only spirometry results in the top three quality grades were included in the analysis. | Total: 24,995  Acceptable: 15,084 | Pre-BD FEV_1_/FVC<0.70 in subjects <70 years  Pre-BD FEV_1_/FVC<0.65 in subjects aged 70-80 years  Pre-BD FEV_1_/FVC<0.60 in subjects aged >80 years | 4231 cases  FEV_1_ % predicted  ≥80%: 951 (22.5%)  50-80%: 2217 (52.4%)  30-50%: 818 (19.3%)  <30%: 245 (5.8%) |
| **Løkke**  **2012**[**^25^**](#_ENREF_25) | Eligible: 4000  Invited: NR  Data on 4049 subjects with complete data  Mean age: 57.5%*  Male: 51%  Smoking status  Current: 59%  Former: 37%  Never: 4% | Device: NR  Bronchodilator: 0.4mg salbutamol for those with initial airflow obstruction  Operator: NR  Standard: Danish Respiratory Society guidelines. Aimed to achieve at least three forced expiratory manoeuvres and at least two measurements differing <5%  Quality control: NR | Total: 4049  Acceptable: NR | Pre-BD FEV_1_/FVC≤0.7 & post-BD FEV_1_ showing <200mL improvement or a negative corticosteroid reversibility test (spirometry performed 6 weeks after administration of 1600ug budesonide daily or 37.5mg oral prednisolone daily for 14 days) for those with an FEV_1_ improvement of 200-500mL | 878 cases  FEV_1_ % predicted  ≥80%: 307* (35%)  50-79%: 439* (50%)  <50%: 132* (15%) |
| **Price**  **2006**[**^27^**](#_ENREF_27) | Eligible: NR  Invited: 17,361  Subjects with acceptable spirometry:  Mean age: 58.2  Male: 49.3%  Smoking status  Current: 44.5%  Former: 55.5% | Device: EasyOne spirometer (ndd Medical Technologies)  Bronchodilator: 2.5mg salbutamol/albuterol  Operator: NR  Standard: ATS standards  Quality control: Principal investigators conducted blinded review of all spirometry loops. A pulmonologist not associated with the study reviewed all loops on which there was disagreement. | Total: 898  Acceptable: 818 | Post-BD FEV_1_/FVC<0.7 | 155 cases  FEV_1_ % predicted  NR |
| **de Queiroz**  **2012**[**^28^**](#_ENREF_28) | Eligible: NR  Invited: 350  Subjects with acceptable spirometry:  Mean age: 65  Male: NR  Smoking status  NR | Device: Spirotrac® (Vitalograph)  Bronchodilator: NR  Operator: NR  Standard: Brazilian Thoracic Association  Quality control: NR | Total: 214  Acceptable: 200 | Post-BD FEV_1_/FVC<0.7 | 45 cases  FEV1% predicted:  ≥80%: 22 (48.9%)  50-80%: 17 (37.8%)  30-50%: 6 (13.3%)  <30%: 0 |
| **Sandelowsky 2011**[**^29^**](#_ENREF_29) | Eligible: 250  Invited: 190  Subjects with acceptable spirometry:  Mean age: 55  Male: 44.2%  Smoking status  Current: 52.9%  Former: 47.1% | Device: Vitalograph Alpha®  Bronchodilator: 8µg formoterol  Operator: NR  Standard: NR  Quality control: NR | Total: 150  Acceptable: 138 | Post-BD FEV_1_/FVC<0.7  (Used ECCS reference equations for disease severity) | 38 cases  FEV_1_% predicted:  ≥80%: 17 (44.7%)  50-80%: 20 (52.6%)  30-50%: 1 (2.6%)  <30%: 0 |
| **Sichletidis**  **2011**[**^30^**](#_ENREF_30) | Eligible: 1250  Invited: 1250  Subjects with acceptable spirometry  Mean age: 65.3  Male: 57.1%  Smoking status  Current/ex: 48.8%  Never: 51.2% | Device: Vitalograph  Bronchodilator:400µg salbutamol  Operator: Pulmonary specialists  Standard: ATS/ERS standards  Quality control: Spirometry performed and interpreted by pulmonary specialists according to ATS/ERS standards | Total: 1250  Acceptable: 1078 | Post-BD FEV_1_/FVC<0.7 | 111 cases  FEV_1_ % predicted  ≥80%:40 (36.0%)  50-80%:53 (47.7%)  30-50%:16 (14.4%)  <30%:2 (1.8%) |
| **Stratelis**  **2004**[**^31^**](#_ENREF_31) | Eligible: 5332  Invited: 5332  Subjects who performed spirometry:  Mean age: 48  Male: 43%  Smoking status  NR | Device: Flowscreen version 3.10gb, Vitalograph Compact II, Vitalograph Alpha, Vicatest P2a  Bronchodilator: None  Operator: Trained COPD nurses  Standard: ATS standards  Quality control: Spirometry results were re-evaluated by an experienced physician. Spirometry judged not to be optimal or showing evidence of obstruction was performed again by the physician. | Total: 512  Acceptable: NR | Pre-BD FEV_1_/VCmax<88% predicted for males and <89% predicted for females | 141 cases  FEV_1_% predicted:  ≥80%: 95 (63.3%)  50-80%: 52 (34.7%)  30-50%: 3 (2.0%)  <30%: 0  (NB. Reported numbers do not summate to 141) |
| **Takahashi**  **2003**[**^32^**](#_ENREF_32) | Eligible: NR  Invited: NR  Subjects with acceptable spirometry:  Mean age: 63.1  Male: 76.3%  Smoking status  Current: 41.7%  Former: 29.8%  Never: 28.5% | Device: Peakman-8, Chest Corp  Bronchodilator: None  Operator: Trained physician  Standard: Subjects were asked to perform at least three forced expiratory manoeuvres. No standard reported.  Quality control: Spirometry data were reviewed by pulmonary specialists | Total: 1168  Acceptable: 1040 | Pre-BD FEV_1_/FVC<0.7 | 281 cases  FEV_1_% predicted:  ≥80%: 62 (22.1%)  50-80%: 60 (21.4%)  30-50%: 30 (10.7%)  <30%: 6 (2.1%) |
| **Takemura**  **2005**[**^33^**](#_ENREF_33) | Eligible: NR  Invited: NR  Subjects aged ≥40 years who performed spirometry:  Mean age: 49.8*  Male: 69.3%  Smoking status  Current: 38.2%  Former: 18.7%  Never: 43.1% | Device: Autospiro AS-505  Bronchodilator: None  Operator: Trained laboratory technologist  Standard: ATS standards  Quality control: Spirometry performed according to ATS standards. No other quality control measures reported. | Total: 10,256  Acceptable: NR  NB. Only including subjects aged ≥40years (80.4% of all subjects) | Pre-BD FEV_1_/VC<0.7 | 425 cases*  FEV_1_% predicted  NR |
| **Thorn**  **2012**[**^34^**](#_ENREF_34) | Eligible: NR  Invited: NR  Subjects who performed spirometry:  Mean age: 61.2  Male: 43.3%  Smoking status  Current/ex: 100% | Device: NR  Bronchodilator: 0.5mg terbutaline  Operator: Nurses  Standard: ATS standards  Quality control: NR | Total: 305  Acceptable: NR | Post-BD FEV_1_/FVC<0.7 | 77 cases  FEV_1_ % predicted  ≥80%: 35 (45.5%)  50-80%: 41 (53.2%)  30-50%: 1 (1.3%)  <30%: 0 |
| **Ulrik**  **2011**[**^35^**](#_ENREF_35) | Eligible: 10,050  Invited: NR  Subjects with complete data (n=3097):  Mean age: 58  Male: 49%  Smoking status  Current: 65%  Former: 32%  Never:3% | Device: NR  Bronchodilator: NR  Operator: NR  Standard: NR  Quality control: NR | Total: 3095  Acceptable: NR | Pre-BD FEV_1_/FVC<0.7 | 1079 cases  FEV_1_% predicted  ≥80%: 302 (28%)*  50-80%: 550 (51%)*  30-50%: 194 (18%)*  <30%: 22 (2%)* |
| **van Schayck 2002**[**^36^**](#_ENREF_36) | Eligible: 229  Invited: 201  Subjects who performed spirometry:  Mean age: NR  Male: NR  Smoking status  Current: 100% | Device: MicroMedical 3300  Bronchodilator: None  Operator: Trained practice assistants with at least three years’ experience (received two 4 hour sessions by a lung function laboratory assistant)  Standard: ATS standards  Quality control: Performance of practice assistants monitored by study author | Total: 201  Acceptable: 169 | Pre-BD FEV_1_% predicted<80% | 30 cases  FEV_1_% predicted  NR |
| **Vandevoorde**  **2007**[**^37^**](#_ENREF_37) | Eligible: 141  Invited: 141  Subjects meeting inclusion criteria (n=146):  Mean age:52.3  Male:42.5%  Smoking status  Current: 100% | Device: Spirobank S, One Flow FVC  Bronchodilator: None  Operator: Participating GPs received spirometry training consisting of a theoretical course followed by two practical sessions in a lung function laboratory.  Standard: ERS standards  Quality control: All spirometry curves were assessed by two study authors. Two other study authors reviewed spirometry curves where there was disagreement. | Total: 129  Acceptable: 121 | Pre-BD FEV_1_/FVC<0.7 | 43 cases  FEV_1_% predicted  ≥80%:18 (41.9%)  50-80%:21 (48.8%)  30-50%:4 (9.3%)  <30%:0 |
| **Vrijhoef**  **2003**[**^38^**](#_ENREF_38) | Eligible: NR  Invited: NR  Subjects who performed spirometry:  Mean age: 53.5  Male: 48.5%  Smoking status  Current: 62%  Former: 38% | Device: Vitalograph 2120, Microlab 3300  Bronchodilator: 500µg terbutaline  Operator: Initial spirometry performed by four medical undergraduates trained for a day at a lung function laboratory. Subsequent spirometry performed by a respiratory nurse practitioner.  Standard: ERS standards.  Quality control: NR | Total: 231  Acceptable: NR | Post-BD FEV_1_/FVC<0.65 of predicted ratio or FEV_1_<84% predicted following course of oral prednisolone | 17 cases  FEV1% predicted  NR |

*Value computed from available data

NR=not reported, Age in years

### Table S6 Yield from case finding with spirometry (30 studies)

| **Study** | **Recruitment strategy** | **Mean age**  **(years)** | **Male (%)** | **Smoking**  **status** | **Population** | | **Diagnosed**  **with**  **COPD** | **Subjects diagnosed with COPD**  **as a percentage of those:** | |
| --- | --- | --- | --- | --- | --- | --- | --- | --- | --- |
|  |  |  |  |  | **Eligible** | **Received**  **spirometry** |  | **Eligible** | **Received**  **spirometry** |
| **Al Ghobain 2011** | Opportunistic | 47.9 | 89.6 | Smokers | 1380 | 1380 | 71 | 5.1 | 5.1 |
| **Bednarek 2008** | Active | 56.7 | 39.0 | Any | 2250 | 1986 | 149 | 6.6 | 7.5 |
| **Broekhuizen*** | Opportunistic | 63 | 45 | Any | NR | 353 | 102 | NR | 28.9 |
| **Bunker 2009** | Active | 62.4 | 57 | Smokers | 400 | 79 | 10 | 2.5 | 12.7 |
| **Clotet 2004** | NR | 54 | 80 | Smokers | 177 | 155 | 36 | 20.3 | 23.2 |
| **DeJong 2004** | Active | 55 | 49 | Smokers | NR | 243 | 43 | NR | 17.7 |
| **Freeman 2005** | Active | 61.7 | 52 | Smokers | 1195 | 369 | 62 | 5.2 | 16.8 |
| **Frith 2011** | Active & opportunistic | 61 | 69 | Smokers | 233 | 233 | 57 | 24.5 | 24.5 |
| **Fuller 2012** | Active | 55 | 40 | Any | NR | 185 | 16 | NR | 8.6 |
| **Geijer 2005** | Active | 50 | 100 | Smokers | 918 | 805 | 210 | 22.9 | 26.1 |
| **Hanania 2010** | Opportunistic | NR | 38.1 | Any | NR | 937 | 156 | NR | 16.6 |
| **Kimura 2011a** | Active | 63.3 | 33.7 | Any | 839 | 814 | 59 | 7.0 | 7.2 |
| **Kimura 2011b** | Active | NR | NR | Any | 1300 | 363 | 22 | 1.7 | 6.1 |
| **Kögler 2010*** | Opportunistic | NR | 62.2 | Smokers | NR | 1282 | 516 | NR | 40.2 |
| **Konstantikaki 2011^1^** | Active | 62.5 | 64 | Any | 1084 | 1084 | 76 | 7.0 | 7.0 |
| **Konstantikaki 2011^2^** | Active | 63.5 | 19.6 | Any | NR | 219 | 56 | NR | 25.6 |
| **Kotz 2008** | Active | 52.3 | 58.7 | Smokers | 1052 | 826 | 278 | 26.4 | 33.7 |
| **Leuppi 2010** | Opportunistic | 51.6 | 58.6 | Smokers | 26400 | 24995 | 4231 | 16.0 | 16.9 |
| **Lokke 2012*** | Opportunistic | 57.5 | 51 | Smokers | 4000 | 4049 | 878 | 22.0 | 21.7 |
| **Price 2006** | Active | 58.2 | 49.3 | Smokers | NR | 898 | 155 | NR | 17.3 |
| **Queiroz 2012** | Opportunistic | 65 | NR | Smokers | NR | 214 | 45 | NR | 21.0 |
| **Sandelowsky 2011*** | Active | 55 | 44.2 | Smokers | 250 | 150 | 38 | 15.2 | 25.3 |
| **Sichletidis 2011** | Opportunistic | 65.3 | 57.1 | Any | 1250 | 1250 | 111 | 8.9 | 8.9 |
| **Stratelis 2004** | Active | 48 | 43 | Smokers | 5332 | 512 | 141 | 2.6 | 27.5 |
| **Takahashi 2003** | NR | 63.1 | 76.3 | Any | NR | 1168 | 281 | NR | 24.1 |
| **Takemura 2005** | Opportunistic | 49.8 | 69.3 | Any | NR | 10256 | 425 | NR | 4.1 |
| **Thorn 2012** | Opportunistic | 61.2 | 43.3 | Smokers | NR | 305 | 77 | NR | 25.2 |
| **Ulrik 2011*** | Opportunistic | 58 | 49 | Smokers | 10050 | 3095 | 1079 | 10.7 | 34.9 |
| **Vandevoorde 2007** | Opportunistic | 52.3 | 42.5 | Smokers | 141 | 129 | 43 | 30.5 | 33.3 |
| **Vrijhoef 2003** | Opportunistic | 53.5 | 48.5 | Smokers | NR | 231 | 17 | NR | 7.4 |
| **Van Schayck** | Opportunistic | NR | NR | Smokers | 229 | 201 | 30 | 13.1 | 14.9 |

1. Open spirometry programme, 2. Case-finding programme, NR=not reported

*Only recruited subjects complaining of respiratory symptoms or an acute respiratory infection

**Table S7 Results: studies evaluating** screening questionnaires

| **Study** | **Population** | **Questionnaire** | **Spirometry** | **Screened** | **Definition of COPD** | **New cases of COPD** |
| --- | --- | --- | --- | --- | --- | --- |
| **Buffels**  **2004**[**^4^**](#_ENREF_4) | Eligible: 3158  Invited: 3158  Data on subjects with spirometry  Mean age: NR  Male: 45%  Smoking status  Current: 30.7%  Former: 18.1%  Never: 50.1% | Items:  Cough >2 weeks  Dyspnoea during mild exercise or at night  Nasal allergy or hay fever  Visit to doctor for wheeze or chronic cough  Threshold:  ≥1 symptom | Device: Spirobank spirometer with Winspiro software  Bronchodilator: None  Operator: GPs who had received 12 hours of training  Standard: NR  Quality control: Technical support provided to GPs throughout the study. Accuracy of GP-performed spirometry for a patient was compared to result from a lab technician. | Questionnaire  Total: 3158  Positive: 728  Spirometry  Total: 703  Acceptable: NR | Pre-BD FEV_1_/FVC<88.5% predicted for men & <89.3% for women | With symptoms and airflow obstruction:  126 cases  FEV_1_ % predicted  (all spirometry tests)  >80%: 53 (39%)  50-80%: 69 (51%)  30-50%: 12 (9%)  ≤30%: 1 (<1%) |
| **Castillo**  **2009**[**^6^**](#_ENREF_6) | Eligible: NR  Invited: 254  Data on subjects who completed questionnaire  Mean age: 55  Male: 42%  Smoking status  Current/ex: 77%  Never: 23% | Items:  More breathlessness than people of same age  Chronic cough  Chronic sputum  Age >40 years  Smoking  Threshold:  ≥3 positive items | Device: EasyOne Spirometer (ndd Medical Technologies)  Bronchodilator: None  Operator: Pharmacists who had attended a four-day spirometry training course.  Standard: ERS/ATS standards  Quality control: All spirometry curves reviewed by a lung function expert. Subjects with FEV_1_/FVC<0.7 referred to lung function unit for spirometry. | Questionnaire  Total: 161  Positive: 100  Spirometry  Total: 96  Acceptable: 86 | Pre-BD FEV_1_/FVC<0.7 | 21 cases  Severity:  Mild: 13 (61.9%)  Moderate: 7 (33.3%)  Severe: 1 (4.8%)  (Severity grading criteria not reported) |
| **Dirven**  **2013a**[**^9^**](#_ENREF_9)  **-Patient-managed arm** | Eligible: 6,393  Invited: 6,393  Data on subjects who completed questionnaire  Mean age: 53.5  Male: 51.0%  Smoking status  Current: 40%  Former: 12%  Never: 48% | COPD Diagnostic Question (referred to as “Respiratory Health Screening Questionnaire”)  Items:  See Price 2006  Threshold:  Score >19.5 | Device: NR  Bronchodilator: NR  Operator: NR  Standard: Dutch College of General Practitioners  Quality control: NR | Questionnaire  Total: 1715  Positive: 186  Spirometry  Total: 140  Acceptable: NR | Post-BD FEV_1_/FVC<0.7 & physician’s clinical evaluation | 25 cases  FEV_1_ % predicted  NR |
| **Dirven**  **2013a**[**^9^**](#_ENREF_9)  **-Practice-managed arm** | Eligible: 3,715  Invited: 3,715  Data on subjects who completed questionnaire  Mean age: 53.7  Male: 49.7%  Smoking status  Current: 44%  Former: 14%  Never: 42% | As above | As above | Questionnaire  Total: 1855  Positive: 251  Spirometry  Total: 135  Acceptable quality: NR | As above | 48 cases  FEV_1_ % predicted  NR |
| **Dirven**  **2013b**[**^10^**](#_ENREF_10) | Eligible: 849  Invited: 831  Mean age: NR  Male: NR  Smoking status:  Current: NR  Former: NR  Never: NR | As above (Dirven 2013a) | Device: NR  Bronchodilator: NR  Operator: NR  Standard: NR  Quality control: NR | Questionnaire  Total: 293  Positive: 50  Spirometry  Total: 39  Acceptable: NR | Post-BD FEV_1_/FVC<0.7 & physician’s clinical evaluation | 9 cases  FEV_1_ % predicted  NR |
| **Freeman**  **2005**[**^12^**](#_ENREF_12) | Eligible: 1195  Invited: 1195  Data on subjects with spirometry  Mean age: 61.7  Male: 52%  Smoking status  Current: 54.1%  Former: 45.9% | Multiple response questionnaire  Items:  Age  Smoking status  Pack-years  Cough  Dyspnoea  Wheeze  Threshold:  NR (included only “best” reported) | Spirometer: Micro-Med handheld spirometer with Spida software  Bronchodilator: 5mg salbutamol for those with prior respiratory medication/history of asthma/FEV_1_<80% predicted  Operator: Trained respiratory nurse  Standard: ATS standards. Minimum of 3 tests or until results within 5%.  Quality control: All spirometry results reviewed by a physician to ensure compliance with ATS standards | Questionnaire  Total: 369  Positive: 121*  Spirometry  Total: 369  Acceptable: NR | Post-BD FEV_1_/FVC<0.7 and lack of reversibility (reversibility defined as increase in FEV_1_ of ≥200mL and 15% from pre-BD FEV_1_  (not clear if all were post-BD) | 47 cases*  FEV_1_ % predicted NR |
| **Freeman**  **2005**[**^12^**](#_ENREF_12) | Eligible: 1195  Invited: 1195  Data on subjects with spirometry  Mean age: 61.7  Male: 52%  Smoking status  Current: 54.1%  Former: 45.9% | Binary response questionnaire  As above | As above | Questionnaire  Total: 369  Positive: 142*  Spirometry  Total: 369  Acceptable: NR | As above | 54 cases*  FEV_1_ % predicted NR |
| **Frith**  **2011**[**^13^**](#_ENREF_13) | Eligible: 233  Invited: 237  Data on subjects with acceptable spirometry:  Mean age: 61  Male: 69%  Smoking status  Current: 45%  Former: 55%  Never: <1% | COPD Diagnostic Questionnaire  Items:  See Price 2006  Threshold:  ≥19.5 | Device: EasyOne spirometer (NDD)  Bronchodilator: 360mcg salbutamol  Operator: trained operators using ATS/ERS guidelines  Standard: ATS/ERS standards. At least 3 adequate baseline and post-BD FVC manoeuvres performed.  Quality control: spirometry quality monitored by a respiratory physiologist blinded to the questionnaire and Piko-6® results | Questionnaire  Total: 233  Positive: 107*  Spirometry  Total: NR  Acceptable: 204 | Post-BD FEV_1_/FVC<0.7 | 40 cases*  FEV_1_ % predicted see table 2 |
| **Frith**  **2011**[**^13^**](#_ENREF_13) | As above | As above  Threshold:  ≥16.5 | As above | Questionnaire  Total: 233  Positive: 163*  Spirometry  As above | As above | 52 cases*  FEV_1_ % predicted see table 2 |
| **Hanania**  **2010**[**^16^**](#_ENREF_16) | Eligible: NR  Invited: NR  Data on 837 subjects with spirometry:  Mean age: NR  Male: 38.1%  Smoking status NR | Lung Function Questionnaire  Items:  Age  Cough  Wheeze  Dyspnoea  Smoking  Threshold:  Score ≤18 | Device: EasyOne spirometers (ndd Medical Technologies)  Bronchodilator: None  Operator: NR  Standard: NR  Quality control: Investigators rated spirometry quality based on reliability and reproducibility. Only included traces considered reliable. | Questionnaire  Total: 937  Positive: 537*  Spirometry  Total: 937  Acceptable: NR  Analysed: 837 | Pre-BD FEV_1_/FVC<0.7 | 129 cases*  FEV_1_ % predicted  See table 2 |
| **Haroon**  **2013**[**^17^**](#_ENREF_17) | Eligible: 815  Invited: 813  Data on subjects who returned questionnaire  Mean age: 53.0  Male: 60.8%  Smoking status Current: 62.3%  Former: 45.8% | Postal questionnaire  Items  Cough  Wheeze  Sputum  Dyspnoea  Smoking  Occupational exposures  Demographics  Threshold  ≥1 chronic respiratory symptom | Device: Microloop and Micro GP spirometers with Spida 5 software  Bronchodilator: None  Operator: Trained practice nurses  Standard: ATS/ERS standards  Quality control: First five spirometry results were quality checked by a respiratory scientist | Questionnaire  Total: 212  Positive: 166  Spirometry  Total: 70  Acceptable: NR | Pre-BD FEV_1_/FVC<0.7 with FEV_1_<80% predicted, lack of reversibility (reversibility defined as increase in FEV_1_ of 200mL and 15% from pre-BD FEV1) and presence of respiratory symptoms. | 10 cases  FEV_1_ % predicted  50-80%: 14 (87.5%)  30-50%: 2 (12.5%)  <30%: 0 (0%)  (NB. FEV1 % predicted includes results from both study arms) |
| **Haroon**  **2013**[**^17^**](#_ENREF_17) | Eligible: 819  Invited: 258  Mean age: 55.3  Male: 67.6%  Smoking status Current: 55.9%  Former: 54.1% | Opportunistic questionnaire  As above | As above | Questionnaire  Total: 111  Positive: 81  Spirometry  Total: 28  Acceptable: NR | As above | 6 cases  FEV_1_ % predicted  As above |
| **Kotz**  **2008**[**^22^**](#_ENREF_22) | Eligible: 1052  Invited: 1052  Data on subjects with spirometry:  Mean age: 52.3  Male: 58.7%  Smoking status  Current: 100% | COPD Diagnostic Questionnaire (CDQ)  Items:  See Price 2006  Threshold  ≥19.5 | Device: Vitalograph 2120  Bronchodilator: 500 µg terbutaline  Operator: Two qualified research assistants under supervision of a pulmonologist.  Standard: ATS/ERS standards  Quality control: All spirometry results were validated by a pulmonologist and specialised lung function laboratory assistant not involved in the trial- both were blinded to questionnaire scores. | Questionnaire  Total: 1052  Positive: 554*  Spirometry  Total: 826  Acceptable: 716 | Post-BD FEV_1_/FVC<0.7 | 233 cases*  FEV_1_ % predicted  See table 2 |
| **Kotz**  **2008**[**^22^**](#_ENREF_22) | As above | As above  Threshold  ≥16.5 | As above | Questionnaire  Total: 1052  Positive: 844*  Spirometry  As above | As above | 316 cases*  FEV_1_ % predicted  See table 2 |
| **Laniado-Laborin**  **2011**[**^23^**](#_ENREF_23) | Eligible: NR  Invited: NR  Data on 2293 subjects with acceptable spirometry:  Mean age: 57.6  Male: 39.8%  Smoking status  Current: 42.1%*  Former: NR  Never: NR | Items  Smoking  Exposure to biomass smoke and/or dusts  Cough  Sputum production  Dyspnoea  Threshold  ≥1 symptom or risk factor | Device: EasyOne spirometer (ndd Medical Technologies)  Bronchodilator: NR  Operator: Certified respiratory therapist  Standard: ATS/ERS standards  Quality control: Poor quality results were excluded from analysis. | Questionnaire  Total: NR  Positive: NR  Spirometry  Total: 2617  Acceptable: 2293 | Post-BD FEV_1_/FVC<0.7  (Used reference equations from NHANES III for Mexican Americans) | 472 cases  FEV_1_ % predicted  ≥80%: 96 (20.3%)  50-79%: 217 (46.0%)  30-49%: 123 (26.1%)  <30%: 36 (7.6%) |
| **Mintz**  **2011**[**^26^**](#_ENREF_26) | Eligible: NR  Invited: 4956  Data on subjects who completed questionnaire:  Mean age: 53.9*  Male: 51.2%  Smoking status  Current: 57.6%  Former: 42.4% | Lung Function Questionnaire  Items  See Hanania 2010  Threshold  Score ≤18 | Device: Biomedical Systems, St Louis, MO  Bronchodilator: 360µg albuterol  Operator: Trained site staff  Standard: ATS standards  Quality control: Only data collected from acceptable spirometry manoeuvres were included. Patients producing unacceptable spirometry were allowed to repeat this within seven days of study visit. | Questionnaire  Total: 1575  Positive: 1228*  Spirometry  Total: 1225  Acceptable: 849 | LFQ≤18 & post-BD FEV_1_/FVC<0.7 | 315 cases*  NB. restricted to subjects ≥40 years  FEV_1_ % predicted NR |
| **Price**  **2006**[**^27^**](#_ENREF_27) | Eligible: NR  Invited: 17,361  Data on subjects with acceptable spirometry  Mean age: 58.2  Male: 49.3%  Smoking status  Current: 44.5%  Former: 55.5% | COPD Diagnostic Questionnaire  Items  Age  Smoking pack-years  Weather-affected cough  Cough up phlegm in absence of a cold  Early morning cough  Wheeze  Allergies  Threshold  ≥19.5 | Device: EasyOne spirometer (NDD)  Bronchodilator:2.5mg salbutamol/albuterol  Operator: NR  Standard: ATS standards  Quality control: Principal investigators conducted blinded review of all spirometry loops. A pulmonologist not associated with the study reviewed all loops on which there was disagreement. | Questionnaire  Total: 898  Positive: 243*  Spirometry  Total: 898  Acceptable: 818 | Post-BD FEV_1_/FVC<0.7 | 91 cases*  FEV_1_ % predicted  See table 2 |
| **Price**  **2006**[**^27^**](#_ENREF_27) | As above | As above  Threshold  ≥16.5 | As above | Questionnaire  Total: 898  Positive: 406*  Spirometry  As above | As above | 125 cases*  FEV_1_ % predicted  NR |
| **Sichletidis 2011**[**^30^**](#_ENREF_30) | Eligible: 1250  Invited: 1250  Data on subjects with acceptable spirometry:  Mean age: 65.3  Male:57.1%  Smoking status  Current/ex: 48.8%  Never: 51.2% | COPD Diagnostic Questionnaire (referred to as “International Primary Airways Group Questionnaire”)  Items  See Price 2006  Threshold  Score ≥17 | Device: Vitalograph  Bronchodilator:400µg salbutamol  Operator: Pulmonary specialists  Standard: ATS/ERS standards  Quality control: Spirometry performed and interpreted by pulmonary specialists according to ATS/ERS standards | Questionnaire  Total: 1250  Positive: 693*  Spirometry  Total: NR  Acceptable: 1078 | Post-BD FEV_1_/FVC<0.7 | 103 cases *  FEV_1_ % predicted  See table 2 |

ATS=American Thoracic Society, ERS=European Respiratory Society, LFQ=Lung Function Questionnaire

**Table S8 Results: studies evaluating handheld flow meters**

| **Study** | **Population** | **Handheld spirometer** | **Spirometry** | **Screened** | **Definition of COPD** | **New cases of COPD** |
| --- | --- | --- | --- | --- | --- | --- |
| **Duong-Quy**  **2009**[**^11^**](#_ENREF_11) | Eligible: 2464  Invited: NR  Data on 2397 screened subjects  Mean age: 52  Male: 99.7%  Smoking status  Current: 88.9%  Former: 11.1% | Pre-BD Piko-6®  Best of three manoeuvres selected.  All measures where FEV_1_/FEV_6_>1 were excluded.  Operator: NR  Threshold  FEV_1_/FEV_6_<0.7 | Device: SpiroLab II  Bronchodilator: short-acting β_2_-agonist (specific drug not specified)  Operator: NR  Standard: ATS/ERS standards.  Quality control: Required at least three measures and at least two within 150mL | Handheld flow meter  Total: 2464  Positive: 324  Spirometry  Total:144  Acceptable: NR | Post-BD FEV_1_/FVC<0.7 with <200mL or 12% reversibility | 136 cases  FEV_1_ % predicted  <80%: 65  50-79%: 63  30-49%: 8  <30%: 0 |
| **Frith**  **2011**[**^13^**](#_ENREF_13) | Eligible: 233  Invited: 237  Data on subjects with acceptable spirometry:  Mean age: 61  Male: 69%  Smoking status  Current: 45%  Former: 55%  Never: <1% | Pre-BD Piko-6®  Operator: Study nurse or GP  Threshold:  FEV_1_/FEV_6_<0.75 (optimal cut-point) | Device: EasyOne spirometer (ndd Medical Technologies)  Bronchodilator: 360mcg salbutamol  Operator: trained operators  Standard: ATS/ERS standards. At least 3 adequate baseline and post-BD FVC manoeuvres performed.  Quality control: spirometry quality monitored by a respiratory physiologist blinded to the questionnaire and Piko-6® results | Handheld flow meter  Total: 233  Positive: 97*  Spirometry  Total: NR  Acceptable: 204 | Post-BD FEV_1_/FVC<0.7 | 46 cases*  FEV_1_ % predicted: see table 2 |
| **Kaufmann 2009**[**^18^**](#_ENREF_18) | Eligible: NR  Invited: NR  Data on subjects who used handheld flow meter:  Mean age: 54.8*  Male: 37.7%  Smoking status  Current: 30.4%  Former: 4.1%  Never: 65.5% | Pre-BD Piko-6®  Operator: trained study nurses  Threshold: FEV_1_/FEV_6_<80% | Device: NR  Bronchodilator: Combivent (dose not reported)  Operator: Trained study nurses  Standard: NR  Quality control: Study nurses were trained in a pulmonary function laboratory | Handheld flow meter  Total: 507  Positive: 106  Spirometry  Total: 74  Acceptable: NR | Reversibility of FEV_1_≤200mL or <15% after administration of Combivent (dose NR) | 14 cases  FEV_1_ % predicted  ≥80%: 9 (64.3%)  50-80%: 5 (35.7%)  <50%: 0  (NB. Study also included five patients with normal spirometry as having COPD) |
| **Sichletidis 2011**[**^30^**](#_ENREF_30) | Eligible: 1250  Invited: 1250  Data on subjects with acceptable spirometry:  Mean age:65.3  Male:57.1%  Smoking status  Current/ex: 48.8%  Never: 51.2% | Post-BD Piko-6®  Operator: GPs who had two hours training  Bronchodilator: 400µg salbutamol  Threshold  Post-BD FEV_1_/FEV_6_<0.7 | Device: Vitalograph  Bronchodilator:400µg salbutamol  Operator: Pulmonary specialists  Standard: ATS/ERS standards  Quality control: Spirometry performed and interpreted by pulmonary specialists according to ATS/ERS standards | Handheld flow meter  Total: 1250  Positive: 147*  Spirometry  Total: NR  Acceptable: 1078 | Post-BD FEV_1_/FVC<0.7 | 89 cases*  FEV_1_ % predicted  See table 2 |
| **Sichletidis 2011**[**^30^**](#_ENREF_30) | As above | Post-BD Piko-6® and COPD Diagnostic Questionnaire (see table 3) | As above | Handheld flow meter & screening questionnaire  Total: 1250  Positive: 111*  Spirometry:  As above | As above | 82 cases*  FEV_1_ % predicted  See table 2 |
| **Thorn**  **2012**[**^34^**](#_ENREF_34) | Eligible: NR  Invited: NR  Data on subjects with spirometry:  Mean age: 61.2  Male:43.3%  Smoking status  Current/ex: 100% | Pre-BD COPD 6®  Operator: Nurses  Threshold FEV_1_/FVC<0.73 | Device: NR  Bronchodilator: 0.5mg terbutaline  Operator: Nurses  Standard: ATS standards  Quality control: NR | Handheld flow meter  Total: 305  Positive: 106*  Spirometry  Total: 305  Acceptable: NR | Post-BD FEV_1_/FVC<0.7 | 61 cases*  FEV_1_ % predicted  See table 2 |

ATS=American Thoracic Society, ERS=European Respiratory Society, NR=not reported

### Table S9 Yield from case finding with screening questionnaires and/or handheld flow meter

| **Study** | **Recruitment**  **strategy** | **Mean age**  **(years)** | **Male (%)** | **Smoking**  **status** | **Population** | | | **COPD** | **Subjects diagnosed with COPD**  **as a percentage of those:** | | |
| --- | --- | --- | --- | --- | --- | --- | --- | --- | --- | --- | --- |
|  |  |  |  |  | **Eligible** | **Screened** | **Received**  **spirometry** |  | **Eligible** | **Screened** | **Received**  **spirometry** |
| **Screening questionnaires** | | | | | | | | | | | |
| ***CDQ (≥19.5)*** | | | | | | | | | | | |
| Dirven 2013a^1^ | Active | 53.5 | 51 | Any | 6393 | 1715 | 140 | 25 | 0.4 | 1.5 | 17.9 |
| Dirven 2013a^2^ | Active | 53.7 | 49.7 | Any | 3715 | 1855 | 135 | 48 | 1.3 | 2.6 | 35.6 |
| Dirven 2013b | Active | NR | NR | Any | 849 | 293 | 39 | 9 | 1.1 | 3.1 | 23.1 |
| Frith 2011 | Active & opportunistic | 61 | 69 | Smokers | 233 | 233 | 107 | 40 | 17.2 | 17.2 | 37.4 |
| Kotz 2008 | Active | 52.3 | 58.7 | Smokers | 1052 | 1052 | 554 | 233 | 22.1 | 22.1 | 42.1 |
| Price 2006 | Active | 58.2 | 49.3 | Smokers | NR | 898 | 243 | 91 | NR | 10.1 | 37.4 |
| ***CDQ (≥16.5)*** | | | | | | | | | | | |
| Frith 2011 | Active & opportunistic | 61 | 69 | Smokers | 233 | 233 | 163 | 52 | 22.3 | 22.3 | 31.9 |
| Kotz 2008 | Active | 52.3 | 58.7 | Smokers | 1052 | 1052 | 844 | 316 | 30.0 | 30.0 | 37.4 |
| Price 2006 | Active | 58.2 | 49.3 | Smokers | NR | 898 | 406 | 125 | NR | 13.9 | 30.8 |
| Sichletidis 2011 | Opportunistic | 65.3 | 57.1 | Any | 1250 | 1250 | 693 | 103 | 8.2 | 8.2 | 14.9 |
| ***All other questionnaires*** | | | | | | | | | | | |
| Buffels 2004 | Opportunistic | NR | 45 | Any | 3158 | 3158 | 703 | 126 | 4.0 | 4.0 | 17.9 |
| Castillo 2009 | Opportunistic | 55 | 42 | Any | NR | 161 | 96 | 21 | NR | 13.0 | 21.9 |
| Freeman 2005^3^ | Active | 61.7 | 52 | Smokers | 1195 | 369 | 121 | 47 | 3.9 | 12.7 | 38.8 |
| Freeman 2005^4^ | Active | 61.7 | 52 | Smokers | 1195 | 369 | 142 | 54 | 4.5 | 14.6 | 38.0 |
| Hanania 2010 | Opportunistic | NR | 38.1 | Any | NR | 937 | 537 | 129 | NR | 13.8 | 24.0 |
| Haroon 2013 | Opportunistic | 55.3 | 67.6 | Smokers | 819 | 111 | 28 | 6 | 0.7 | 5.4 | 21.4 |
| Haroon 2013 | Active | 53.0 | 60.8 | Smokers | 815 | 212 | 70 | 10 | 1.2 | 4.7 | 14.3 |
| Laniado-Laborin 2011 | Active | 57.6 | 39.8 | Any | NR | NR | 2617 | 472 | NR | NR | 18.0 |
| Mintz 2011 | NR | 53.9 | 51.2 | Smokers | NR | 1575 | 1225 | 315 | NR | 20.0 | 25.7 |
|  | | | | | | | | | | | |
| **Handheld flow meter** | | | | | | | | | | | |
| Duong-Quy 2009 | Active | 52 | 99.7 | Smokers | 2464 | 2464 | 144 | 136 | 5.5 | 5.5 | 94.4 |
| Frith 2011 | Active & opportunistic | 61 | 69 | Smokers | 233 | 233 | 97 | 46 | 19.7 | 19.7 | 47.4 |
| Kaufmann 2009 | Opportunistic | 54.8 | 37.7 | Any | NR | 507 | 74 | 14 | NR | 2.8 | 18.9 |
| Sichletidis 2011 | Opportunistic | 65.3 | 57.1 | Any | 1250 | 1250 | 147 | 89 | 7.1 | 7.1 | 60.5 |
| Thorn 2012 | Opportunistic | 61.2 | 43.3 | Smokers | NR | 305 | 106 | 61 | NR | 20.0 | 57.5 |
|  | | | | | | | | | | | |
| **CDQ & handheld flow meter** | | | | | | | | | | | |
| Sichletidis 2011 | Opportunistic | 65.3 | 57.1 | Any | 1250 | 1250 | 111 | 82 | 6.6 | 6.6 | 73.9 |

1. Patient-managed arm, 2. Practice-managed arm, 3. Multiple response questions, 4. Binary response questions

CDQ=COPD Diagnostic Questionnaire (score threshold), NR=not reported

## References

1. Al Ghobain M, Al-Hajjaj MS, Wali SO. Prevalence of chronic obstructive pulmonary disease among smokers attending primary healthcare clinics in Saudi Arabia. *Ann Saudi Med* 2011;31(2):129-33.

2. Bednarek M, Maciejewski J, Wozniak M, Kuca P, Zielinski J. Prevalence, severity and underdiagnosis of COPD in the primary care setting. *Thorax* 2008;63(5):402-07.

3. Broekhuizen BD, Sachs AP, Hoes AW, Moons KG, van den Berg JW, Dalinghaus WH, et al. Undetected chronic obstructive pulmonary disease and asthma in people over 50 years with persistent cough. *The British journal of general practice : the journal of the Royal College of General Practitioners* 2010;60(576):489-94.

4. Buffels J, Degryse J, Heyrman J, Decramer M, Study D. Office spirometry significantly improves early detection of COPD in general practice: the DIDASCO Study. *Chest* 2004;125(4):1394-9.

5. Bunker J, Hermiz O, Zwar N, Dennis SM. Feasibility and efficacy of COPD case finding by practice nurses. *Aust Fam Physician* 2009;38(10):826-30.

6. Castillo D, Guayta R, Giner J, Burgos F, Capdevila C, Soriano JB, et al. COPD case finding by spirometry in high-risk customers of urban community pharmacies: A pilot study. *Respiratory medicine* 2009;103(6):839-45.

7. Clotet J, Gomez-Arbones X, Ciria C, Albalad JM. Spirometry is a good method for detecting and monitoring chronic obstructive pulmonary disease in high-risk smokers in primary health care. *Arch Bronconeumol* 2004;40(4):155-59.

8. DeJong SR, Veltman RH. The effectiveness of a CNS-led community-based COPD screening and intervention program. *Clinical nurse specialist CNS* 2004;18(2):72-9.

9. Dirven JAM, Tange HJ, Muris JWM, van Haaren KMA, Vink G, van Schayck OCP. Early detection of COPD in general practice: patient or practice managed? A randomised controlled trial of two strategies in different socioeconomic environments. *Prim Care Resp J* 2013;22(3):331-37.

10. Dirven JAM, Tange HJ, Muris JWM, van Haaren KMA, Vink G, van Schayck OCP. Early detection of COPD in general practice: implementation, workload and socioeconomic status. A mixed methods observational study. *Prim Care Resp J* 2013;22(3):338-43.

11. Duong-Quy S, Hua-Huy T, Mai-Huu-Thanh B, Doan-Thi-Quynh N, Le-Quang K, Nguyen-Van H, et al. [Early detection of smoking related chronic obstructive pulmonary disease in Vietnam]. *Revue des maladies respiratoires* 2009;26(3):267-74.

12. Freeman D, Nordyke RJ, Isonaka S, Nonikov DV, Maroni JM, Price D, et al. Questions for COPD diagnostic screening in a primary care setting. *Respiratory medicine* 2005;99(10):1311-8.

13. Frith P, Crockett A, Beilby J, Marshall D, Attewell R, Ratnanesan A, et al. Simplified COPD screening: validation of the PiKo-6(R) in primary care. *Primary care respiratory journal : journal of the General Practice Airways Group* 2011;20(2):190-8, 2 p following 98.

14. Fuller L, Conrad WF, Heaton PC, Panos R, Eschenbacher W, Frede SM. Pharmacist-managed chronic obstructive pulmonary disease screening in a community setting. *Journal of the American Pharmacists Association : JAPhA* 2012;52(5):e59-66.

15. Geijer RMM, Sachs APE, Hoes AW, Salome PL, Lammers JWJ, Verheij TJM. Prevalence of undetected persistent airflow obstruction in male smokers 40-65 years old. *Fam Pract* 2005;22(5):485-89.

16. Hanania NA, Mannino DM, Yawn BP, Mapel DW, Martinez FJ, Donohue JF, et al. Predicting risk of airflow obstruction in primary care: Validation of the lung function questionnaire (LFQ). *Respiratory medicine* 2010;104(8):1160-70.

17. Haroon S, Adab P, Griffin C, Jordan R. Case finding for chronic obstructive pulmonary disease in primary care: a pilot randomised controlled trial. *Brit J Gen Pract* 2013;63(606).

18. Kaufmann M, Hartl S, Geyer K, Breyer MK, Burghuber OC. Measuring FEV6 for Detecting Early Airway Obstruction in the Primary Care Setting. *Respiration* 2009;78(2):161-67.

19. Kimura K, Kurosaki H, Wakabayashi R, Motegi T, Ishii T, Yamada K, et al. Concerns with the Health Check-up System for Chronic Obstructive Pulmonary Disease on two Japanese Islands. *Internal Med* 2011;50(19):2135-41.

20. Kogler H, Metzdorf N, Glaab T, Welte T. Preselection of patients at risk for COPD by two simple screening questions. *Respiratory medicine* 2010;104(7):1012-19.

21. Konstantikaki V, Kostikas K, Minas M, Batavanis G, Daniil Z, Gourgoulianis KI, et al. Comparison of a network of primary care physicians and an open spirometry programme for COPD diagnosis. *Respiratory medicine* 2011;105(2):274-81.

22. Kotz D, Nelemans P, van Schayck CP, Wesseling GJ. External validation of a COPD diagnostic questionnaire. *The European respiratory journal* 2008;31(2):298-303.

23. Laniado-Laborin R, Rendon A, Bauerle O. Chronic obstructive pulmonary disease case finding in Mexico in an at-risk population. *Int J Tuberc Lung D* 2011;15(6):818-23.

24. Leuppi JD, Miedinger D, Chhajed PN, Buess C, Schafroth S, Bucher HC, et al. Quality of Spirometry in Primary Care for Case Finding of Airway Obstruction in Smokers. *Respiration* 2010;79(6):469-74.

25. Lokke A, Ulrik CS, Dahl R, Plauborg L, Dollerup J, Kristiansen LC, et al. Detection of previously undiagnosed cases of COPD in a high-risk population identified in general practice. *Copd* 2012;9(5):458-65.

26. Mintz ML, Yawn BP, Mannino DM, Donohue JF, Hanania NA, Grellet CA, et al. Prevalence of airway obstruction assessed by lung function questionnaire. *Mayo Clinic proceedings* 2011;86(5):375-81.

27. Price DB, Tinkelman DG, Nordyke RJ, Isonaka S, Halbert RJ, Group CQS. Scoring system and clinical application of COPD diagnostic questionnaires. *Chest* 2006;129(6):1531-9.

28. de Queiroz MCDAM, Moreira MAC, Rabahi MF. Underdiagnosis of COPD at primary health care clinics in the city of Aparecida de Goiania, Brazil. *J Bras Pneumol* 2012;38(6):692-99.

29. Sandelowsky H, Stallberg B, Nager A, Hasselstrom J. The prevalence of undiagnosed chronic obstructive pulmonary disease in a primary care population with respiratory tract infections - a case finding study. *Bmc Fam Pract* 2011;12.

30. Sichletidis L, Spyratos D, Papaioannou M, Chloros D, Tsiotsios A, Tsagaraki V, et al. A combination of the IPAG questionnaire and PiKo-6(R) flow meter is a valuable screening tool for COPD in the primary care setting. *Primary care respiratory journal : journal of the General Practice Airways Group* 2011;20(2):184-9, 1 p following 89.

31. Stratelis G, Jakobsson P, Molstad S, Zetterstrom O. Early detection of COPD in primary care: screening by invitation of smokers aged 40 to 55 years. *Brit J Gen Pract* 2004;54(500):201-06.

32. Takahashi TU, Ichinose M, Inoue H, Shirato K, Hattori T, Takishima T. Underdiagnosis and undertreatment of COPD in primary care settings. *Respirology* 2003;8(4):504-08.

33. Takemura H, Hida W, Sasaki T, Sugawara T, Sen T. Prevalence of chronic obstructive pulmonary disease in Japanese people on medical check-up. *Tohoku J Exp Med* 2005;207(1):41-50.

34. Thorn J, Tilling B, Lisspers K, Jorgensen L, Stenling A, Stratelis G. Improved prediction of COPD in at-risk patients using lung function pre-screening in primary care: a real-life study and cost-effectiveness analysis. *Primary care respiratory journal : journal of the General Practice Airways Group* 2012;21(2):159-66.

35. Ulrik CS, Lokke A, Dahl R, Dollerup J, Hansen G, Cording PH, et al. Early detection of COPD in general practice. *International journal of chronic obstructive pulmonary disease* 2011;6:123-27.

36. van Schayck CP, Loozen JMC, Wagena E, Akkermans RP, Wesseling GJ. Detecting patients at a high risk of developing chronic obstructive pulmonary disease in general practice: cross sectional case finding study. *Brit Med J* 2002;324(7350):1370-73.

37. Vandevoorde J, Verbanck S, Gijssels L, Schuermans D, Devroey D, De Backer J, et al. Early detection of COPD: A case finding study in general practice. *Respiratory medicine* 2007;101(3):525-30.

38. Vrijhoef HJM, Diederiks JPM, Wesseling GJ, Van Schayck CP, Spreeuwenberg C. Undiagnosed patients and patients at risk for COPD in primary health care: early detection with the support of non-physicians. *J Clin Nurs* 2003;12(3):366-73.
